# The PlMYB73–PlMYB70–PlMYB108 complex regulates *PlTPS1* to promote geraniol biosynthesis in *Paeonia lactiflora*

**DOI:** 10.1093/hr/uhaf141

**Published:** 2025-05-29

**Authors:** Qian Zhao, Yuqing Li, Lina Gu, Yehua Yang, Di He, Jianrang Luo, Yanlong Zhang

**Affiliations:** College of Landscape Architecture and Arts, Northwest A&F University, Yangling 712100, China; National Engineering Research Center for Oil Peony, Yangling 712100, China; College of Landscape Architecture and Arts, Northwest A&F University, Yangling 712100, China; National Engineering Research Center for Oil Peony, Yangling 712100, China; College of Landscape Architecture and Arts, Northwest A&F University, Yangling 712100, China; National Engineering Research Center for Oil Peony, Yangling 712100, China; College of Landscape Architecture and Arts, Northwest A&F University, Yangling 712100, China; National Engineering Research Center for Oil Peony, Yangling 712100, China; College of Landscape Architecture and Arts, Northwest A&F University, Yangling 712100, China; National Engineering Research Center for Oil Peony, Yangling 712100, China; College of Landscape Architecture and Arts, Northwest A&F University, Yangling 712100, China; National Engineering Research Center for Oil Peony, Yangling 712100, China; College of Landscape Architecture and Arts, Northwest A&F University, Yangling 712100, China; National Engineering Research Center for Oil Peony, Yangling 712100, China

## Abstract

Geraniol contributes significantly to the floral scent of herbaceous peony (*Paeonia lactiflora*) and is abundant in fragrant cultivars. However, the regulatory mechanism of geraniol biosynthesis in herbaceous peony remains unclear. In this study, we identified a transcriptional regulatory complex (PlMYB73–PlMYB70–PlMYB108) that cooperatively regulated geraniol biosynthesis in herbaceous peony. The three MYB members were identified through correlation analysis between geraniol content and gene expression profiles in 17 herbaceous peony cultivars. Transient overexpression and gene silencing experiments revealed that PlMYB73, PlMYB108, and PlMYB70 positively regulated *PlTPS1* expression and geraniol accumulation. PlMYB108 and PlMYB70 directly upregulate *PlTPS1* by binding to the TAACCA and CAACTG motifs, respectively, as demonstrated by yeast one-hybrid, dual-luciferase, and electrophoretic mobility shift assays. Although PlMYB73 did not directly bind to the *PlTPS1* promoter, yeast two-hybrid, bimolecular fluorescence complementation, luciferase complementation imaging, and dual-luciferase assays revealed its interaction with PlMYB70 in the nucleus, resulting in synergistic activation of *PlTPS1*. PlMYB108 was also found to interact with PlMYB70. The three MYB transcription factors formed the PlMYB73–PlMYB70–PlMYB108 complex. Gene co-overexpression and co-silencing experiments demonstrated that the complex significantly enhanced geraniol biosynthesis. In conclusion, our research provides novel insights into the molecular mechanism by which transcription factors cooperatively regulate geraniol biosynthesis.

## Introduction

Geraniol, an acyclic monoterpenoid released from the flowers of diverse plant species, is widely used in the cosmetic and fragrance industries due to its pleasant rose-like odor [1]. Studies have shown that geraniol can attract pollinators [2]. Geraniol also mediates plant–environment interactions and responses to abiotic and biotic stresses [3–5]. Therefore, a deeper understanding of geraniol biosynthesis and its regulatory mechanisms will pave the way for enhancing floral scent and producing economically valuable terpenoids.

In plants, geraniol is primarily synthesized through the terpene synthase (TPS)-catalyzed pathway. This canonical route involves the conversion of geranyl diphosphate (GPP) to geraniol by geraniol synthase, a member of the TPS gene family [6]. GPP is generated in plastids via the methylerythritol 4-phosphate (MEP) pathway, in which GPP synthase (GPPS) condenses isopentenyl diphosphate and dimethylallyl diphosphate as substrates [7]. TPS enzymes catalyze the formation of terpene skeletons from prenyl diphosphate precursors such as GPP and farnesyl diphosphate (FPP), and are central to terpenoid biosynthesis [8]. Phylogenetically, plant TPS genes are classified into seven subfamilies (TPS-a to TPS-g). The TPS-b subfamily is mainly responsible for monoterpene biosynthesis, while certain TPS-g members also contribute to this process [9]. To date, geraniol synthases belonging to the TPS-b and TPS-g subfamilies have been functionally characterized in multiple plant species, such as *Citrus sinensis* [1], *Camellia sinensis* [10], *Dendrobium officinale* [3], and *Gardenia jasminoides* [11]. In addition to the plastidial TPS-mediated synthesis, an atypical geraniol synthesis pathway in the cytoplasm has been reported in *C. sinensis* [5, 12], *Rosa hybrida* [13], and *Rosa chinensis* [14]. This pathway converts GPP to geraniol via Nudix hydrolase and petal-derived phosphatase. It has been confirmed that geraniol in herbaceous peony (*Paeonia lactiflora*) is synthesized by geraniol synthase, which is classified into the TPS-g subfamily [15]. Despite advances in characterizing key structural genes, the transcriptional regulatory framework governing geraniol biosynthesis remains poorly understood and requires further study.

Terpenoid biosynthesis is regulated by the expression levels of TPS genes, which are in turn controlled by transcription factors (TFs) [16, 17]. The diversity of TPS genes makes the transcriptional regulation of terpenoid biosynthesis more complex compared with other secondary metabolite pathways [18]. Recent studies have identified TF families, including WRKY, NAC, bHLH, MYB, AP2/ERF, and bZIP, that regulate terpenoid biosynthesis in plants [19]. These TFs can activate or inhibit gene expression in response to environmental stimuli or developmental signals, thus coordinating terpenoid biosynthesis [20, 21]. Among them, the MYB family has been studied more extensively [20]. However, most of the previous studies have focused on the transcriptional regulation of nonvolatile terpenoids, such as tanshinones in *Salvia miltiorrhiza* [22–25], triterpenoids in *Betula platyphylla* [26], carotenoids in *Solanum lycopersicum* [27] and *Medicago truncatula* [28], and paclitaxel in *Taxus chinensis* [29]. The release of volatile terpenoids plays a significant role in both ornamental value and the ecological interactions of plants [18]. Recent advances in floral scent research have identified MYB TFs that regulate the biosynthesis of volatile terpenoids [30]. RcMYB1 enhances monoterpene production in *R. hybrida* [31]. AmMYB24 can bind to the promoter of the ocimene synthase gene *AmOCS* in *Antirrhinum majus* to promote ocimene production [32]. MYB TFs that regulate volatile compound release have also been reported in *Mentha spicata* [33], *Osmanthus fragrans* [34], *Hedychium coronarium* [35], *Lilium* [36, 37], and *Curcuma alismatifolia* [38]. MYB TFs can regulate volatile terpenoid biosynthesis independently or by forming complexes with other TFs [39]. The MYB-bHLH complex has been extensively studied and characterized in *Freesia hybrida* [40], *Arabidopsis thaliana* [40], *C. sinensis* [41], and *Chrysanthemum* [42]. To our knowledge, no MYB-MYB complexes that regulate terpenoid biosynthesis have been reported.

Herbaceous peony is a popular ornamental flower with more than a thousand cultivars that are characterized by large and colorful flowers [43]. Due to its ornamental value, herbaceous peony is increasingly used as cut flower material for arrangements or bouquets, with broad market prospects [44]. The attractive floral scent of herbaceous peony is a key ornamental and ecological trait that attracts insect pollinators [43]. We previously identified several strongly fragrant herbaceous peony cultivars and demonstrated that monoterpenoids are the dominant floral scent components. Moreover, geraniol is an abundant and characteristic floral scent component in fragrant herbaceous peony cultivars, substantially contributing to their fragrance [43]. The cultivar ‘Wu Hua Long Yu’ (WHLY) was selected due to its high scores in ornamental and sensory evaluations. It is characterized by an elegant flower shape and a rich, pleasant floral scent, making it an ideal material for floral scent research [15]. Monoterpenoids are the dominant volatiles released by ‘WHLY’ during full bloom. Geraniol (45.03%) and its derivatives (16.43%) collectively constitute 61.46% of the total monoterpenes, serving as key pollinator attractants [15]. Geraniol derivatives (citronellol and citral) are derived from geraniol and play roles in plant defense, environmental interactions, and physiological regulation [45–47]. Thus, understanding geraniol biosynthetic regulation is critical for enhancing floral scent in herbaceous peony. To investigate geraniol biosynthesis, we identified seven TPS genes from ‘WHLY’ flowers, among which *PlTPS1* was demonstrated to specifically catalyze geraniol biosynthesis [15]. However, the regulatory mechanism of geraniol biosynthesis remains to be further studied.

In this study, three R2R3-MYB TFs, PlMYB73 (subgroup 22), PlMYB108 (subgroup 20), and PlMYB70 (subgroup 22), were identified from transcriptome data. These proteins formed a transcriptional regulatory complex that activated *PlTPS1* expression, thereby promoting geraniol biosynthesis. Our study provides new insights into the molecular mechanisms of terpenoid production and lays a foundation for improving the regulatory pathways of geraniol biosynthesis.

## Results

### Transcriptome analysis revealed three candidate R2R3-MYB TFs that regulated geraniol biosynthesis in *P. lactiflora*

To investigate the molecular mechanisms of geraniol biosynthesis in *P. lactiflora* ‘WHLY’, we selected five flower developmental stages (S1–S5; Fig. S1A) to analyze geraniol release patterns via headspace solid phase microextraction-gas chromatography–mass spectrometry (HS-SPME-GC–MS). There was no geraniol release from flowers at the S1 and S2 stages, while a peak was detected at S3 followed by a decline during senescence (S4–S5; Fig. S1A). Since geraniol release was highest at S3, we further divided S3-stage plants into seven organs (pistil, stamen, torus, calyx, petal, stem, and leaf; Fig. S1B). The results showed that geraniol was mainly released from petals, with small amounts from stamens, tori, and calyces, but undetected in other organs (Fig. S1B).

**Figure 1:**
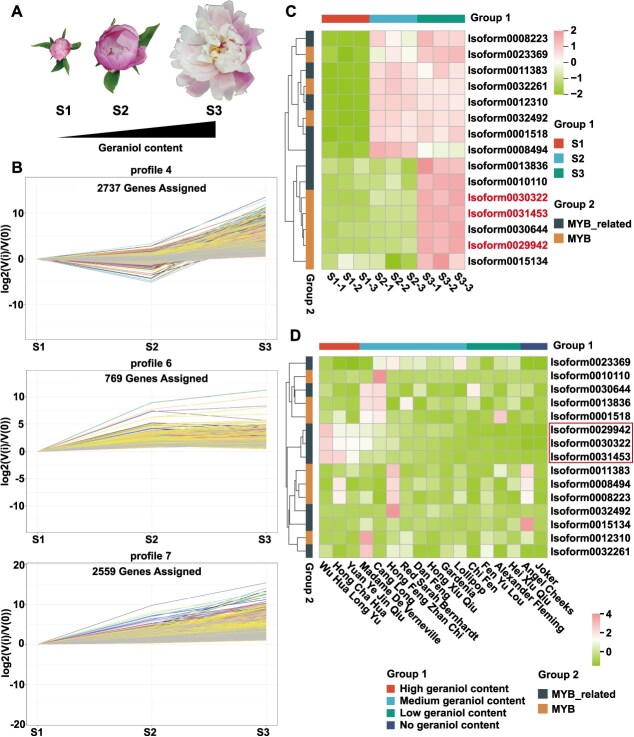
Identification of MYB TFs involved in geraniol biosynthesis in *P. lactiflora* ‘WHLY’. (A) The three developmental stages of ‘WHLY’ flowers. (B) Expression profiles associated with geraniol release patterns. (C) RPKM values of 15 MYB TFs from transcriptome data. RPKM values (*z-*score) are represented by color gradations. (D) Expression patterns of 15 MYB TFs across 17 cultivars. Gene expression levels (*z-*score) are represented by color gradations.

Subsequently, transcriptome analysis was performed on petals at the S1, S2, and S3 stages (Fig. 1A). Considering the strong association between MYB TFs and terpenoid biosynthesis [18], we screened MYB TFs in the transcriptome data. In this study, we employed Short Time-series Expression Miner (STEM) analysis to identify three trend profiles (4, 6, 7) associated with geraniol release patterns in *P. lactiflora* ‘WHLY’ (Fig. 1B). After removing redundant sequences, 15 MYB TFs in these three profiles were identified as potentially involved in geraniol biosynthesis (Fig. 1C).

In our previous study, we performed HS-SPME-GC–MS on 17 herbaceous peony cultivars at the S3 stage (Fig. S1C). Figure S1D shows the geraniol content in these cultivars, which were categorized as follows: three with high content (>1000 ng·g^−1^), eight with medium content (200–1000 ng·g^−1^), four with low content (100–200 ng·g^−1^), and two with no detectable content [43]. We quantified the expression levels of 15 MYB TFs across the 17 cultivars using reverse transcription-quantitative polymerase chain reaction (RT-qPCR). Three MYB TFs (Isoform0029942, Isoform0030322, and Isoform0031453) that showed a strong correlation with geraniol release patterns were ultimately selected (Fig. 1D, Fig. S2).

**Figure 2:**
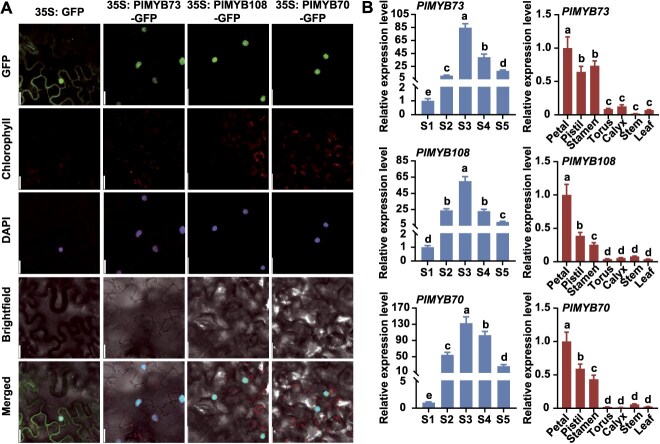
Subcellular localization and relative expression levels of three MYB TFs. (A) Subcellular localization of PlMYB73, PlMYB108, and PlMYB70. Scale bar: 20 μm. (B) Relative expression levels of *PlMYB73*, *PlMYB108*, and *PlMYB70* across five stages and seven organs. *GAPDH* served as the internal reference gene. Data are presented as mean ± SD from three biological replicates. Different lowercase letters above columns indicate statistically significant differences (*P* < 0.05).

Protein sequence alignment analysis showed that all three MYB members were R2R3-MYB TFs with R2 and R3 domains at the N-terminus (Fig. S3A). Phylogenetic analysis was performed using the three R2R3-MYB TFs and *A. thaliana* R2R3-MYB TFs (Table S1). One R2R3-MYB TF (Isoform0030322) in subgroup 20 and two R2R3-MYB TFs (Isoform0029942, Isoform0031453) in subgroup 22 were identified (Fig. S3B). Isoform0029942 showed the highest homology to *A. thaliana* MYB73, and was thus named PlMYB73. Isoform0030322 and Isoform0031453 showed the highest homology to *A. thaliana* MYB108 and MYB70, respectively, and were designated PlMYB108 and PlMYB70. Subcellular localization analysis revealed that PlMYB73, PlMYB108, and PlMYB70 were localized in the nuclei of *Nicotiana benthamiana* leaf cells (Fig. 2A).

Expression levels of these MYB TFs across developmental stages were analyzed, with transcript abundance peaking at the S3 stage. Their expression trends correlated with the geraniol release pattern observed across the five stages (Fig. 2B, Fig. S1A). Furthermore, organ-specific expression analysis revealed that all three MYB TFs exhibited peak transcript levels in petals, and their expression patterns aligned with the spatial geraniol release pattern (Fig. 2B, Fig. S1B).

### Silencing and overexpression of *PlMYB73, PlMYB108,* and *PlMYB70* in *P. lactiflora-*affected geraniol biosynthesis

To verify the function of PlMYB73, PlMYB108, and PlMYB70 in geraniol biosynthesis, we silenced or overexpressed the three MYB TFs in *P. lactiflora* ‘WHLY’ petals using tobacco rattle virus (TRV)-based virus-induced gene silencing (VIGS) and *Agrobacterium*-mediated transient overexpression, respectively. Empty vectors corresponding to each construct were used as negative controls. Petal discs expanded within 3 days after infiltration and were then sampled (Fig. S4). Silenced or overexpressed petal discs were identified by PCR (Fig. S5), and PCR-positive samples were further analyzed via RT-qPCR and HS-SPME-GC–MS.

Previous studies have quantified the expression levels of structural genes in the MEP pathway, among which *PlDXS2*, *PlDXS3*, *PlGPPS1*, and *PlGPPS3*, associated with the floral scent release pattern, were identified as key factors in monoterpene release [48]. In addition, *PlTPS1* was shown to be the only geraniol synthase in herbaceous peony that catalyzes GPP into geraniol [15].

**Figure 3:**
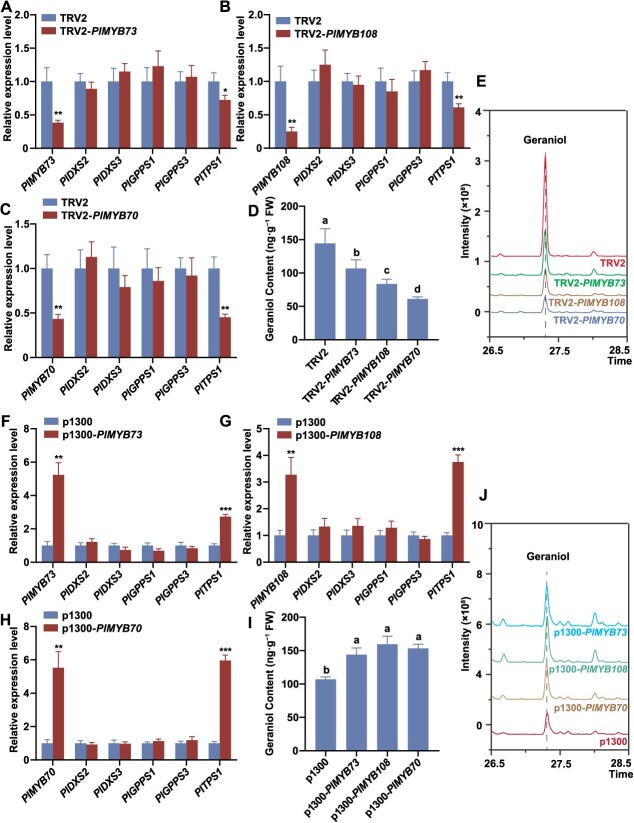
Functional analysis of PlMYB73*,* PlMYB108, and PlMYB70 in *P. lactiflora* ‘WHLY’. (A) RT-qPCR analysis of petal discs infiltrated with empty TRV2 vector and TRV2-*PlMYB73*. (B) RT-qPCR analysis of petal discs infiltrated with empty TRV2 vector and TRV2-*PlMYB108*. (C) RT-qPCR analysis of petal discs infiltrated with empty TRV2 vector and TRV2-*PlMYB70*. (D) Changes in geraniol content after silencing three MYB TFs. (E) Detection of geraniol in *PlMYB73/108/70*-silenced petal discs and the control via HS-SPME-GC–MS. (F) RT-qPCR analysis of petal discs infiltrated with empty p1300 vector and p1300-*PlMYB73*. (G) RT-qPCR analysis of petal discs infiltrated with empty p1300 vector and p1300-*PlMYB108*. (H) RT-qPCR analysis of petal discs infiltrated with empty p1300 vector and p1300-*PlMYB70*. (I) Changes in geraniol content after overexpressing three MYB TFs. (J) Detection of geraniol in *PlMYB73/108/70*-overexpressed petal discs and the control via HS-SPME-GC–MS. The mass spectrum and chemical structure of geraniol are shown in Fig. S6. *GAPDH* served as the internal reference gene. Data are presented as mean ± SD from three biological replicates. Different lowercase letters above columns indicate statistically significant differences (*P* < 0.05). Significant differences (*P < 0.05; **P < 0.01; ***P < 0.001) were observed between TRV2 and TRV2-*PlMYB73/108/70*, and between p1300 and p1300-*PlMYB73/108/70*.

Gene silencing significantly reduced the expression levels of *PlMYB73*, *PlMYB108*, and *PlMYB70* compared with the control. However, this suppression did not significantly alter the expression of *PlDXS2*, *PlDXS3*, *PlGPPS1*, or *PlGPPS3* (Fig. 3A–C). Silencing these three MYB TFs significantly reduced *PlTPS1* expression (Fig. 3A–C) and decreased geraniol content (Fig. 3D–E). In contrast, overexpression of the three MYB TFs significantly upregulated *PlTPS1* expression and increased geraniol content (Fig. 3F–J). These results demonstrated that PlMYB73, PlMYB108, and PlMYB70 promoted geraniol biosynthesis in herbaceous peony by upregulating *PlTPS1* expression.

**Figure 4:**
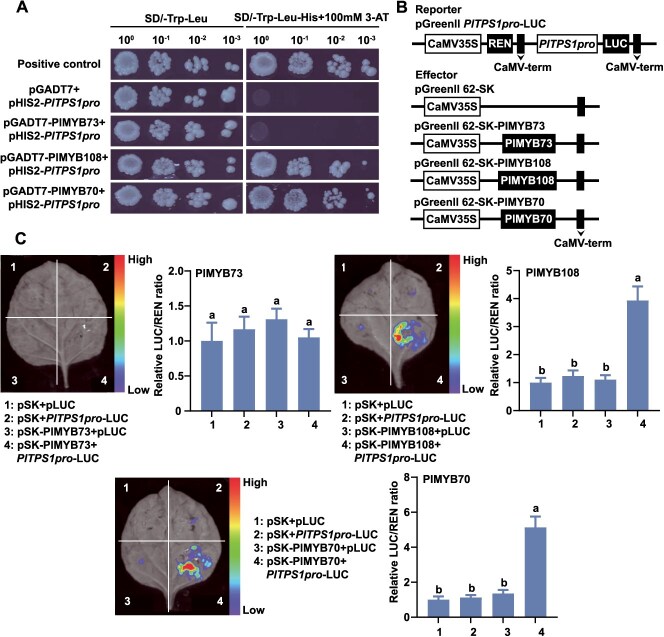
PlMYB108 and PlMYB70 directly bind to *PlTPS1pro* and affect its activity. (A) Interactions between TFs (PlMYB73, PlMYB108, and PlMYB70) and *PlTPS1pro* were detected by Y1H assay. Protein–DNA binding was assessed on SD/−Trp-Leu-His medium containing 3-AT. (B) Vector labeling of the effector and reporter for Dual-LUC assay. CaMV35S, a strong constitutive promoter from cauliflower mosaic virus. CaMV-term, the terminator of CaMV35S. (C) Regulatory effects of TFs (PlMYB73, PlMYB108, and PlMYB70) on *PlTPS1pro* were assessed by Dual-LUC assay. The firefly luciferase (LUC)/*Renilla* luciferase (REN) ratio obtained with the empty vectors (pSK and pLUC) was set to 1. Data are presented as mean ± SD from three biological replicates. Different lowercase letters above columns indicate statistically significant differences (*P* < 0.05).

In previous studies, geraniol derivatives were detected only in the presence of geraniol and were absent when geraniol was not detected [43]. Geraniol derivatives were generated not through TPS catalysis but by geraniol as a substrate [45]. In this research, we analyzed changes in the content of geraniol derivatives (citronellol and citral) after gene silencing or overexpression, and found their changes paralleled those in geraniol (Tables S2 and S3). This indicated that the three MYB TFs may not only regulate geraniol biosynthesis but also influence the levels of geraniol derivatives.

Previous studies have demonstrated that terpenoid compounds, except for geraniol and its derivatives, are synthesized by four TPS genes (*PlTPS4/6/8/9*) in herbaceous peony [15]. To determine whether these MYB TFs regulate other terpenoid biosynthesis, we assessed the expression levels of *PlTPS4/6/8/9* after silencing or overexpression (Fig. S7). Silencing or overexpression of the three MYB TFs did not affect *PlTPS4/6/8/9* expression or other terpenoid production, except for geraniol and its derivatives (Tables S4–S5). These findings indicate that the three MYB TFs specifically regulate geraniol biosynthesis and modulate its derivative content in *P. lactiflora*.

### PlMYB108 and PlMYB70 regulated *PlTPS1* expression in *P. lactiflora*

As PlTPS1 is the most critical enzyme in geraniol biosynthesis, its promoter was cloned by the genome walking method, and a 1776-bp fragment was obtained (Fig. S8). We analyzed the *cis*-acting motifs in the *PlTPS1* promoter (*PlTPS1pro*) sequence. In addition to the basic elements CAAT-box and TATA-box, *PlTPS1pro* contained some hormone and environmental response elements (Fig. S9, Table S6). There were six MYB binding sites in *PlTPS1pro*. To determine whether *PlTPS1* gene was a direct target of PlMYB73, PlMYB108, and PlMYB70, a yeast one-hybrid (Y1H) assay was performed. To determine the minimal 3-AT concentration for suppressing background growth in the Y1H assay, we performed a gradient assay and identified 100 mM as the optimal concentration (Fig. S10). When empty pGADT7 vector, pGADT7-PlMYB73, pGADT7-PlMYB108, or pGADT7-PlMYB70 were co-transformed with pHIS2-*PlTPS1pro*, yeast cells exhibited normal growth on SD/−Trp-Leu medium (Fig. 4A). Only yeast cells harboring pGADT7-PlMYB108 + pHIS2-*PlTPS1pro* and pGADT7-PlMYB70 + pHIS2-*PlTPS1pro* had normal growth on SD/−Trp-Leu-His medium containing 100 mM 3-AT, consistent with the positive control. In contrast, yeast cells expressing pGADT7-PlMYB73 + pHIS2-*PlTPS1pro* failed to grow (Fig. 4A). The results showed that PlMYB108 and PlMYB70 could directly bind to *PlTPS1pro*.

Next, three MYB TFs and *PlTPS1pro* were used to construct dual-luciferase (Dual-LUC) vectors to determine their effects on *PlTPS1pro* activity (Fig. 4B). PlMYB108 and PlMYB70 promoted *PlTPS1pro* activity, showing 3.94-fold and 5.13-fold increases, respectively (Fig. 4C). However, PlMYB73 had no significant effect on *PlTPS1pro* activity. The above results are consistent with those of Y1H assays. These results collectively demonstrate that PlMYB108 and PlMYB70 positively regulate the expression of *PlTPS1*. PlMYB73 had no effect on *PlTPS1pro* activity, suggesting it might interact with other TFs to activate *PlTPS1* expression.

### PlMYB108 and PlMYB70 regulated *PlTPS1* expression to promote geraniol biosynthesis by binding to their corresponding motifs in *PlTPS1pro*

**Figure 5:**
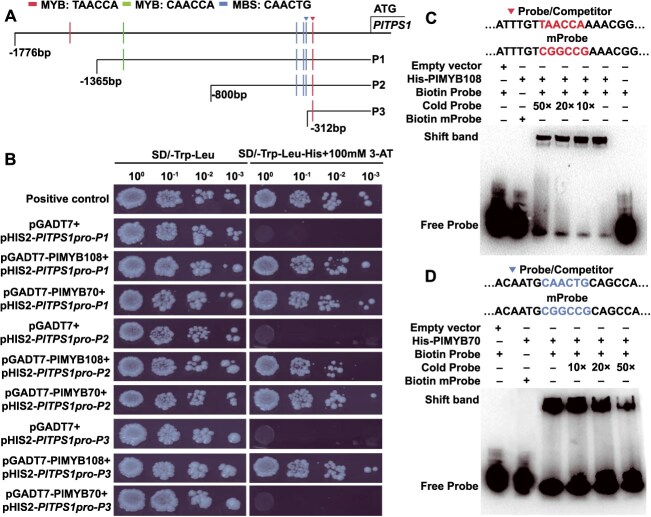
Binding sites of PlMYB108 and PlMYB70 in *PlTPS1pro.* (A) Distribution of MYB binding sites in *PlTPS1pro*, and a schematic diagram of the truncated *PlTPS1pro* fragments*.* (B) Interactions between TFs (PlMYB108/70) and truncated *PlTPS1pro* fragments (P1, P2, and P3) were detected by Y1H assay. Protein-DNA binding was detected on SD/−Trp-Leu-His medium with 3-AT. (C) EMSA-confirmed binding of His-PlMYB108 to the *PlTPS1pro* region containing the TAACCA motif. (D) EMSA-confirmed binding of His-PlMYB70 to the *PlTPS1pro* region containing the CAACTG motif. mProbe, mutant probe. Cold Probe, unlabeled competitor DNA fragment.

The six MYB binding sites in *PlTPS1pro* included two TAACCA motifs, one CAACCA motif, and three CAACTG motifs (Fig. 5A, Fig. S8). To explore the binding sites of PlMYB108 and PlMYB70 within *PlTPS1pro*, we truncated *PlTPS1pro* to lengths of 1365, 800, and 312 bp, and named them P1, P2, and P3, respectively (Fig. 5A). A 3-AT gradient assay was performed, and 100 mM was identified as the minimal concentration required to suppress background growth while maintaining viability of positive controls (Fig. S10). Y1H assays showed that PlMYB108 and PlMYB70 could still bind to *PlTPS1pro* when the promoter was truncated to P1 and P2. However, once the promoter was truncated to 312 bp (P3), only PlMYB108 could bind to *PlTPS1pro* (Fig. 5B). The sole TAACCA motif in P3 may be critical for the binding of PlMYB108 to *PlTPS1pro*. However, PlMYB70 no longer bound to *PlTPS1pro* when the promoter was truncated to P3, suggesting that the PlMYB70 binding site was located in P2. The three MYB binding sites in P2 share the same motif type (CAACTG). Therefore, we hypothesize that the binding motifs of PlMYB108 and PlMYB70 are TAACCA and CAACTG, respectively.

To further confirm this speculation, we performed an electrophoretic mobility shift assay (EMSA). The His-tagged PlMYB108 and PlMYB70, along with the proteins from the empty vector, were successfully purified (Fig. S11). When the labeled probe containing the TAACCA motif was incubated with the control (empty vector), the probe migrated freely without retardation. When the His-PlMYB108 protein was incubated with the labeled TAACCA-containing probe, the protein bound to the probe, resulting in a retarded migration band. The cold probe competed with the labeled probe, reducing His-PlMYB108 binding intensity. The higher the concentration of the cold probe, the stronger the competitive binding. The His-PlMYB108 protein did not bind to the mutated TAACCA probe (Fig. 5C). The above results demonstrated that PlMYB108 directly bound to *PlTPS1pro* through the TAACCA motif. For PlMYB70, the CAACTG motif closest to the *PlTPS1* start codon was selected for EMSA. The results demonstrated direct and specific binding of His-PlMYB70 to this motif, which was abolished by mutation (Fig. 5D). To exclude the possible interaction between MYB TFs and the CAACCA motif, the *PlTPS1pro* region containing the CAACCA motif was selected for EMSA. The results indicated that neither PlMYB108 nor PlMYB70 bound to the CAACCA motif (Fig. S12).

The above findings demonstrated that *PlTPS1* was directly targeted by PlMYB108 and PlMYB70. In summary, the TAACCA and CAACTG motifs closest to the *PlTPS1* start codon played critical roles in the regulation mediated by PlMYB108 and PlMYB70.

### Overexpression of *PlMYB108* and *PlMYB70* in tobacco increased volatile monoterpenoid biosynthesis

To clarify the function of *PlMYB108* and *PlMYB70*, stable expression analysis was carried out in *Nicotiana tabacum*. Positive transgenic tobacco plants were identified by DNA detection, and overexpression lines (OE lines) were obtained (Fig. S13A and B). Relative expression levels of *PlMYB108* and *PlMYB70* in the wild type (WT) and OE lines were quantified by RT-qPCR. *EF1α* served as the internal reference gene. OE-*PlMYB108–*1/2/6 and OE-*PlMYB70*–1/2/4 were selected (Fig. S13C and D). Compared with the WT, OE lines showed no changes in flower color or shape (Fig. S13E). Nonetheless, significant differences were observed in the intensity of floral scent.

**Figure 6:**
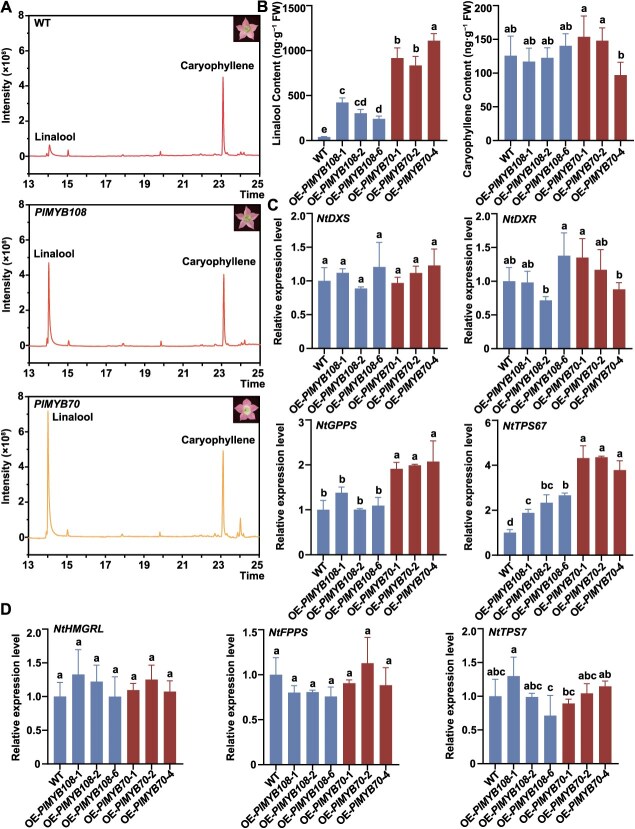
Functional analysis of PlMYB108 and PlMYB70 in *N. tabacum*. (A) The detection of terpenes in the WT, OE-*PlMYB108*, and OE-*PlMYB70* flowers by HS-SPME-GC–MS. (B) Linalool and caryophyllene content in the WT, OE-*PlMYB108*, and OE-*PlMYB70* flowers. (C) Expression levels of genes involved in monoterpenoid biosynthesis in the WT, OE-*PlMYB108*, and OE-*PlMYB70* flowers. (D) Expression levels of genes involved in sesquiterpenoid biosynthesis in the WT, OE-*PlMYB108*, and OE-*PlMYB70* flowers. The mass spectra and chemical structures of linalool and caryophyllene are shown in Fig. S6. *EF1α* served as the internal reference gene. Data are presented as mean ± SD from three biological replicates. Different lowercase letters above columns indicate statistically significant differences (*P* < 0.05).

Volatile terpenoids in the WT and OE flowers were analyzed by HS-SPME-GC–MS. Only two terpenoids, linalool and caryophyllene, were detected (Fig. 6A). OE-*PlMYB108* and OE-*PlMYB70* lines showed 6.18- to 10.84-fold and 21.38- to 28.46-fold higher linalool content than the WT, respectively (Fig. 6A and B). Meanwhile, the expression levels of linalool synthase *NtTPS67* were significantly upregulated in OE-*PlMYB108* and OE-*PlMYB70* lines compared with the WT (Fig. 6C). Furthermore, overexpression of *PlMYB108* did not up- or downregulate the expression of other genes involved in monoterpenoid biosynthesis in tobacco (Fig. 6C). *NtGPPS* expression was specifically upregulated in OE-*PlMYB70* lines (Fig. 6C). However, overexpression of both *PlMYB108* and *PlMYB70* increased linalool but not caryophyllene content (Fig. 6A and B). Genes involved in sesquiterpenoid biosynthesis were not significantly changed in OE-*PlMYB108* and OE-*PlMYB70* lines compared with the WT (Fig. 6D).

In conclusion, overexpression of *PlMYB108* led to an upregulation of *NtTPS67* expression, which resulted in an increase in linalool content. Overexpression of *PlMYB70* upregulated *NtTPS67* and *NtGPPS* expression, promoting linalool biosynthesis. OE-*PlMYB70* lines had higher linalool content and *NtTPS67* expression levels than OE-*PlMYB108* lines. Both MYB TFs were involved in monoterpenoid biosynthesis in tobacco flowers.

### PlMYB70 formed complexes with PlMYB73 and PlMYB108

Previous results showed that PlMYB73 only affected *PlTPS1* expression and was associated with geraniol biosynthesis. However, PlMYB73 did not activate *PlTPS1pro*, suggesting that it might interact with other TFs to activate *PlTPS1* expression. PlMYB108 and PlMYB70 could directly regulate the expression of *PlTPS1* to promote geraniol biosynthesis. Therefore, we investigated all potential pairwise interactions among the three MYB TFs.

**Figure 7:**
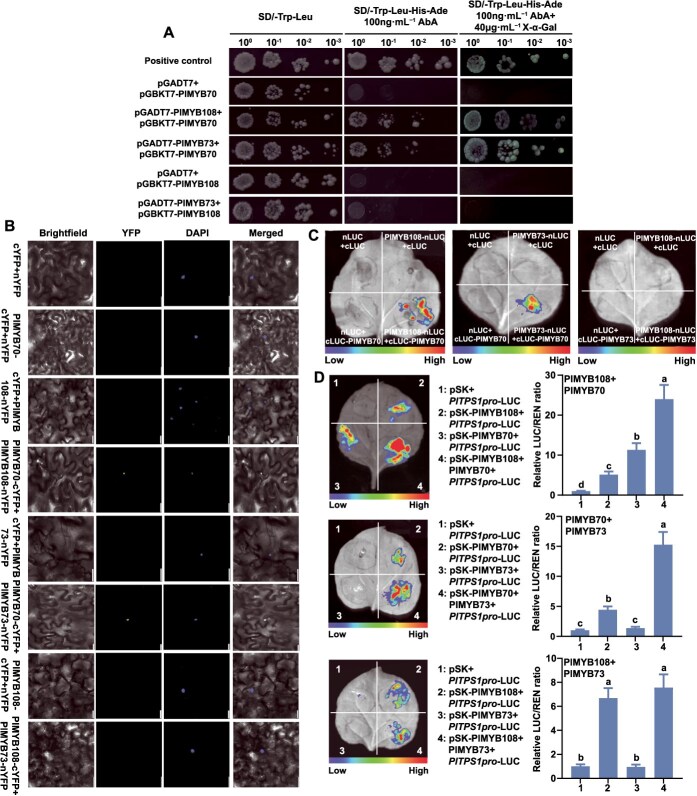
PlMYB73 and PlMYB108 form complexes with PlMYB70 to activate *PlTPS1pro* activity*.* (A) Pairwise interactions between PlMYB70, PlMYB73, and PlMYB108 were detected by Y2H assay. Interactions were detected on SD/−Trp-Leu-His-Ade medium containing X-α-Gal and AbA. (B) Pairwise interactions between PlMYB70, PlMYB73, and PlMYB108 were detected by BiFC assay. (C) Pairwise interactions between PlMYB70, PlMYB73, and PlMYB108 were detected by LCI assay. (D) Regulatory effects of TF interactions on *PlTPS1pro* were analyzed by Dual-LUC assay. The LUC/REN ratio obtained with the empty vectors (pSK and pLUC) was set to 1. Data are presented as mean ± SD from three biological replicates. Different lowercase letters above columns indicate statistically significant differences (*P* < 0.05).

We performed a yeast two-hybrid (Y2H) assay. When pGBKT7-PlMYB73 or pGBKT7-PlMYB108 was co-transformed with the pGADT7-PlMYB70 construct, yeast cells exhibited normal growth and displayed blue coloration in the presence of X-α-Gal and Aureobasidin A (AbA), consistent with the positive control. In contrast, the negative control showed no growth (Fig. 7A). These findings showed that PlMYB70 interacted with PlMYB73 and PlMYB108, but PlMYB73 did not interact with PlMYB108.

Bimolecular fluorescence complementation (BiFC) assay showed that cotransformation of pSPYNE-PlMYB73 or pSPYNE-PlMYB108 with the pSPYCE-PlMYB70 construct led to detectable yellow fluorescent protein (YFP) signals in tobacco leaf cells. Nuclear dye 4′,6-diamidino-2-phenylindole (DAPI) overlapped with YFP signals, indicating nuclear interaction. When pSPYNE-PlMYB73 was cotransformed with the pSPYCE-PlMYB108 construct, no YFP signals could be detected. YFP signals were not detected in other negative controls either (Fig. 7B).

Furthermore, luciferase complementation imaging (LCI) assay showed that the combination of PlMYB73-nLUC + cLUC-PlMYB70 and PlMYB108-nLUC + cLUC-PlMYB70 displayed intense luciferase luminescence in tobacco leaves. The combination of PlMYB73-nLUC + cLUC-PlMYB108 and other negative controls showed no luciferase signals (Fig. 7C).

In summary, these results indicated that PlMYB73 and PlMYB108 formed protein complexes with PlMYB70. Therefore, we conducted a Dual-LUC assay to verify whether the interaction enhanced the activation of *PlTPS1pro*. The results showed that PlMYB73 and PlMYB108 synergistically activated *PlTPS1pro*, with activation levels 4.68- and 2.12-fold higher than those of PlMYB73 and PlMYB108 alone, respectively (Fig. 7D). PlMYB73 alone could not activate *PlTPS1pro*, but its combination with PlMYB70 activated *PlTPS1pro* 3.45-fold higher than that of PlMYB70 alone (Fig. 7D). No interaction was observed between PlMYB73 and PlMYB108, and *PlTPS1pro* activation showed no significant difference between PlMYB108 alone and the combination of PlMYB73 and PlMYB108 (Fig. 7D).

**Figure 8:**
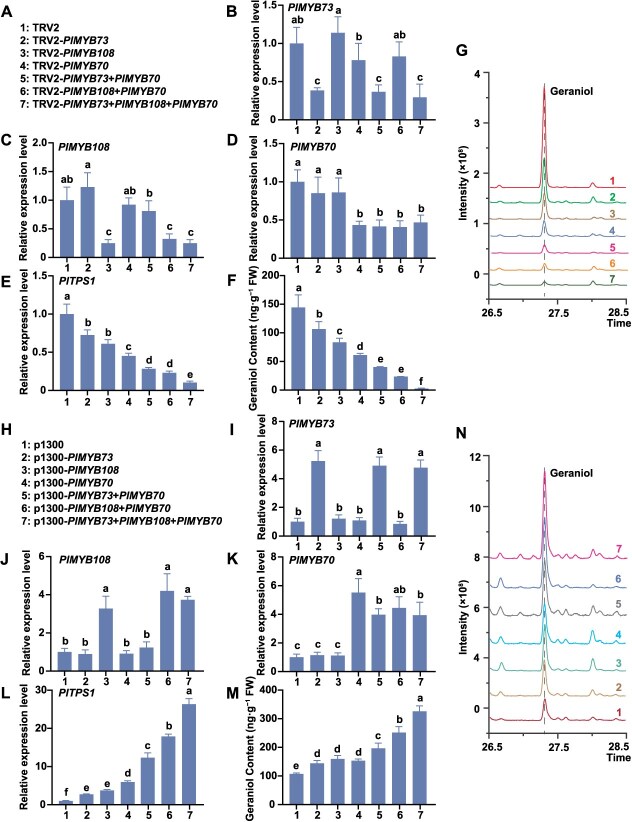
Role of the PlMYB73–PlMYB70–PlMYB108 complex in promoting geraniol biosynthesis in *P. lactiflora* ‘WHLY’. (A) Seven experimental groups for gene silencing. (B) Expression patterns of *PlMYB73* in seven experimental groups after silencing. (C) Expression patterns of *PlMYB108* in seven experimental groups after silencing. (D) Expression patterns of *PlMYB70* in seven experimental groups after silencing. (E) Expression patterns of *PlTPS1* in seven experimental groups after silencing. (F) Geraniol content in seven experimental groups after silencing. (G) Geraniol peak plots in seven experimental groups. (H) Seven experimental groups for gene overexpression. (I) Expression patterns of *PlMYB73* in seven experimental groups after overexpression. (J) Expression patterns of *PlMYB108* in seven experimental groups after overexpression. (K) Expression patterns of *PlMYB70* in seven experimental groups after overexpression. (L) Expression patterns of *PlTPS1* in seven experimental groups after overexpression. (M) Geraniol content in seven experimental groups after overexpression. (N) Geraniol peak plots in seven experimental groups. *GAPDH* served as the internal reference gene. The mass spectrum and chemical structure of geraniol are shown in Fig. S6. Data are presented as mean ± SD from three biological replicates. Different lowercase letters above columns indicate statistically significant differences (*P* < 0.05).

### The PlMYB73–PlMYB70–PlMYB108 complex significantly regulated *PlTPS1* to promote geraniol biosynthesis in *P. lactiflora*

The above results showed that PlMYB73 and PlMYB108 could form complexes with PlMYB70, and these complexes significantly activated *PlTPS1pro*. To further clarify whether PlMYB73, PlMYB70, and PlMYB108 could form a complex and the role of this complex in herbaceous peony, transient silencing or overexpression experiments involving three different combinations were performed in *P. lactiflora* ‘WHLY’ petals. Empty vectors corresponding to each construct were used as negative controls. Petal discs expanded within 3 days after infiltration and were then sampled (Fig. S4). Silenced or overexpressed petal discs were identified by PCR (Fig. S5), and PCR-positive samples were further analyzed via RT-qPCR and HS-SPME-GC–MS.

VIGS experiments were performed with seven experimental groups (Fig. 8A). Furthermore, the corresponding genes were effectively silenced (Fig. 8B–D). After co-silencing *PlMYB73* and *PlMYB70*, *PlTPS1* expression levels and geraniol content were significantly decreased compared with single-MYB TF silencing (Fig. 8E–G). Co-silencing *PlMYB108* and *PlMYB70* also led to a significant decrease in *PlTPS1* expression levels and geraniol content compared with single-MYB TF silencing (Fig. 8E–G). Moreover, among the seven groups, *PlTPS1* expression levels and geraniol content were the lowest in the group with all three MYB TFs silenced (Fig. 8E–G). In contrast, across the seven groups, *PlTPS1* expression levels and geraniol content were the highest in the group that overexpressed all three MYB TFs (Fig. 8H–N). In conclusion, the PlMYB73–PlMYB70–PlMYB108 complex significantly promoted geraniol biosynthesis in herbaceous peony.

In this study, we analyzed changes in geraniol derivatives (citronellol and citral) content after co-silencing or co-overexpression and found that their changes paralleled those in geraniol (Tables S2 and S3). This indicated that the transcription complex might not only regulate geraniol biosynthesis, but also influence the abundance of geraniol derivatives.

## Discussion

Terpenes are the most widely distributed secondary metabolites in plants [49]. Apart from their biological functions, their extensive commercial utilization has resulted in substantial economic advantages [40]. A deeper understanding of regulatory mechanisms governing terpenoid biosynthesis could open new avenues to modify floral scent characteristics and produce industrially important terpenoids. Typically, terpenoid biosynthesis is catalyzed by TPSs through either the mevalonate pathway or the MEP pathway [50]. Within the plant kingdom, genes encoding TPSs frequently exist in multiple copies to facilitate complex metabolic pathways [51], which implies the complexity of terpenoid regulation.

Recent studies have indicated that the transcriptional regulation of terpenoids is distinct from that of other specialized plant metabolites [40]. For instance, the MBW regulatory complex controls anthocyanins and proanthocyanidins [52], whereas flavanols are mainly regulated by MYB TFs alone [53, 54]. However, members of diverse TF families, including AP2/ERF [55, 56], bHLH [23, 57], MYB [58–60], NAC [61, 62], WRKY [63, 64], and bZIP [65, 66], have been shown to regulate terpenoid biosynthesis through direct modulation of structural genes. We hypothesize that the low sequence similarity of TPS genes with identical functions across species may drive the diversity of TFs associated with terpenoid biosynthesis.

Previous studies have shown that many herbaceous peony cultivars emit substantial monoterpenoids, with geraniol and its derivatives comprising the majority [43]. As a critical insect attractant and ornamental trait in herbaceous peony, floral scent is of significant research interest [48]. In *P. lactiflora* ‘WHLY’, geraniol is the dominant floral scent component, peaking during full bloom (S3 stage) and primarily released from petals. Our earlier work identified five TPS genes (*PlTPS1/4/6/8/9*) responsible for terpenoid biosynthesis in herbaceous peony, among which *PlTPS1* uniquely converts GPP to geraniol [15]. Although we have characterized the structural genes, the transcriptional regulation of geraniol biosynthesis remains unclear.

Analysis of gene expression profiles based on transcriptome data is an effective method to study transcriptional regulation of certain traits [40]. To elucidate the regulation of geraniol biosynthesis, we used this approach and identified PlMYB73, PlMYB108, and PlMYB70 (Fig. 1). In our study, both VIGS and transient overexpression experiments demonstrated that PlMYB73, PlMYB108, and PlMYB70 regulated *PlTPS1* expression, thereby influencing geraniol content. To determine whether these three MYB TFs might regulate other TPS genes and consequently influence the biosynthesis of other terpenoids, we analyzed the expression levels of four other TPS genes (*PlTPS4/6/8/9*). The results revealed that neither silencing nor overexpression of each individual MYB TF (PlMYB73, PlMYB108, or PlMYB70) had any detectable effect on the expression of *PlTPS4/6/8/9* or the content of terpenoids synthesized by these four TPSs (Fig. S7, Tables S4 and S5). These findings indicate that PlMYB73, PlMYB108, and PlMYB70 specifically regulate geraniol and its derivatives in herbaceous peony, while the transcriptional regulation of other terpenes involves additional TFs. This further highlights the complexity of transcriptional regulatory networks in plants.

The *Arabidopsis* MYB73 and MYB70 TFs have been studied in multiple plant species, but their roles in terpenoid biosynthesis remain unclear [67, 68]. MYB108-mediated regulation of terpenoid biosynthesis has been reported in *Lilium* [37], *Conyza blinii* [69], and *Artemisia annua* [70]. Our findings demonstrate that PlMYB108 and PlMYB70 directly bind to *PlTPS1pro*, and *PlTPS1* drives geraniol synthesis. The correlation between TF expression levels and geraniol content, alongside silencing/overexpression assays, Y1H, Dual-LUC, and EMSA, collectively supports their roles in regulating *PlTPS1* transcription (Figs 2–5). MYB TFs typically regulate targets by binding specific DNA motifs, with binding specificity varying among MYB members [71]. For example, SlMYB11 binds the TAACCA motif in the *DHAR* promoter [72], while VvMYBA1 recognizes the CAACTG motif in the *VvUFGT1* promoter [73]. In *PlTPS1pro*, six MYB motifs were identified, with PlMYB108 and PlMYB70 binding TAACCA and CAACTG, respectively (Fig. 5). These motifs are closest to the translation start site (Fig. 5). It has been demonstrated that the closer a motif is to the translation start site, the stronger the regulatory effect on gene expression [40].

Considering that a stable transformation system has not been established in herbaceous peony, conducting stable transformation in tobacco serves as a viable method to validate TF functions [57]. It has been found that LaMYC7 regulates the biosynthesis of linalool and caryophyllene in *Lavandula angustifolia*. Similar regulatory effects were achieved through stable transformation in tobacco [57]. In this study, overexpression of *PlMYB108* and *PlMYB70* resulted in increased linalool content in *N. tabacum* flowers (Fig. 6). Additionally, the expression levels of *NtTPS67*, a linalool synthase, were significantly upregulated in *PlMYB108-* and *PlMYB70*-OE lines (Fig. 6). The expression levels of *NtGPPS*, a key structural gene in the MEP pathway, were also significantly upregulated in *PlMYB70*-OE lines (Fig. 6). These findings demonstrate that heterologous expression of PlMYB108 and PlMYB70 regulates linalool biosynthesis in *N. tabacum*.

Transient overexpression of *PlMYB70* and *PlMYB108* specifically increased geraniol content in herbaceous peony, whereas stable overexpression in *N. tabacum* enhanced linalool production. This divergence likely arises from species-specific metabolic network configurations [40]. In herbaceous peony, *PlTPS1* exclusively converts GPP into geraniol, and PlMYB108/70 directly activate *PlTPS1* expression. In contrast, tobacco lacks a *PlTPS1* homolog, and its endogenous linalool synthase *NtTPS67* synthesizes linalool from GPP. The absence of geraniol in tobacco flowers suggests either insufficient GPP allocation or the lack of a functional geraniol synthase.

Species-specific metabolic network architectures and promoter *cis-*acting element variations enable the same TF to perform distinct functions across plant species [40]. This hypothesis is supported by previous studies. For instance, stable transformation of *LaMYC4* from *L. angustifolia* into *A. thaliana* specifically regulates the caryophyllene synthase gene and caryophyllene release. However, its stable expression in tobacco significantly upregulates five terpenoid biosynthesis pathway genes (*NtHMGR*, *NtFPPS*, *NtDXS*, *NtDXR*, and *NtGPPS*), affecting both mono- and sesquiterpenoid biosynthesis in transgenic tobacco flowers [74]. Similarly, MYB21 in *F. hybrida* specifically regulates the linalool synthase gene *FhTPS14*, whereas in *A. thaliana*, it also modulates sesquiterpene synthase genes *AtTPS11* and *AtTPS21*, altering caryophyllene production [40].

The tobacco stable transformation system validated the functional conservation of PlMYB108/70 in monoterpenoid biosynthesis. Notably, PlMYB70 specifically upregulated *NtGPPS* expression, which encodes the enzyme synthesizing GPP for monoterpenoid biosynthesis. This regulatory effect was absent in *P. lactiflora*, potentially due to the unique promoter sequence of *NtGPPS* in *N. tabacum*. The dual regulation of *NtGPPS* and *NtTPS67* by PlMYB70 synergistically enhanced linalool production by simultaneously elevating precursor supply and terminal synthase activity. This indicates that compared with *P. lactiflora*, PlMYB70 exhibits broader regulatory flexibility in *N. tabacum*. Future studies could focus on identifying the *cis-*acting elements in the *NtGPPS* and *NtTPS67* promoters bound by PlMYB108/70 to clarify regulatory divergence between *P. lactiflora* and *N. tabacum*.

Our earlier studies have indicated that *PlDXS2*, *PlDXS3*, *PlGPPS1*, and *PlGPPS3* are key structural genes involved in floral scent emission in *P. lactiflora* [38]. Although PlMYB73 participates in geraniol biosynthesis, it does not affect *PlDXS2*, *PlDXS3*, *PlGPPS1*, or *PlGPPS3* expression, but only influences *PlTPS1* (Fig. 3). Moreover, PlMYB73 cannot bind to or activate *PlTPS1pro*, suggesting that its regulatory role depends on interactions with other TFs (Fig. 4). Current studies have demonstrated that TFs regulating volatile terpenoid biosynthesis can functionally interact within a cooperatively regulatory network [30]. For example, MYC2 and MYB21 form a protein complex regulating terpenoid biosynthesis in *F. hybrida* and *A. thaliana* [40]. CrWRKY1 modulates monoterpene synthase activity by interacting with CrMYC2 [75]. In addition to interacting with TFs from other families, members of the same TF family can form protein complexes to regulate terpenoid biosynthesis. For example, OsTGAP1 and OsbZIP79, both bZIP family members, interact to regulate terpenoid biosynthesis in rice [76, 77]. In this study, PlMYB73 and PlMYB108 formed transcriptional complexes with PlMYB70 in the nucleus, significantly enhancing *PlTPS1* promoter activity (Fig. 7). PlMYB70 served as a critical bridge for the formation of the PlMYB73–PlMYB70–PlMYB108 complex (Figs 7 and 8). Although the functions of MYB70, MYB73, and MYB108 have been studied, few studies have focused on their roles in monoterpenoid biosynthesis, and the interaction among them has not been reported. To our knowledge, this is the first report on MYB-MYB complexes regulating terpenoid biosynthesis.

Among six OE lines, more linalool was released in OE-*PlMYB70* lines than in OE-*PlMYB108* lines. Meanwhile, the expression levels of *NtTPS67* in OE-*PlMYB70* lines were also significantly higher than those in OE-*PlMYB108* lines (Fig. 6). Similar results were found in the VIGS experiment, in which geraniol content and *PlTPS1* expression were significantly lower after *PlMYB70* silencing than those after *PlMYB73* and *PlMYB108* silencing (Fig. 3). In addition, PlMYB70 interacted with both PlMYB73 and PlMYB108 (Fig. 7). Therefore, we speculate that PlMYB70 is the most critical and potent TF regulating terpenoid biosynthesis.

In addition to geraniol, its derivatives (citronellol and citral) are important volatile components in herbaceous peony [43]. Previous studies have demonstrated that citronellol and citral are biosynthesized via the reduction of geraniol, which acts as their essential substrate [44]. Across the five developmental stages, *PlMYB73*, *PlMYB108*, and *PlMYB70* were expressed in stage S2, yet no geraniol was detected (Fig. 2, Fig. S1). In flower organs, despite the expression of these TFs in the pistils, geraniol remained undetectable. In stage S2 and the pistils, geraniol might be synthesized but subsequently converted into citronellol and citral before release. To investigate this, we revisited data from our previous studies on volatile compound emissions in *P. lactiflora* ‘WHLY’. Citronellol and citral were detected both in stage S2 and the pistils, indicating that geraniol could be their precursor. This explains why geraniol was not detected in Stage S2 or the pistils, where these MYB TFs were transcriptionally active. However, the mechanism by which geraniol is converted into citronellol and citral remains to be further studied in herbaceous peony.

Our previous study revealed that geraniol and its derivatives coexist in most herbaceous peony cultivars [43]. Studies have confirmed that geraniol derivatives are released only when geraniol is sufficiently abundant [45]. Heterologous expression of a geraniol synthase gene from *Ocimum basilicum* led to the production of citronellol and other geraniol derivatives [78]. Similarly, Li *et al*. modified *R. hybrida* ‘Juicy Terrazza’ (which was originally unable to release geraniol and its derivatives), and derivatives were detected in the modified plants following the exogenous supply of geraniol and other substrates [45]. Silencing and overexpression experiments showed that geraniol derivative levels paralleled geraniol content changes (Tables S2 and S3). These three MYB TFs regulate the levels of geraniol and its derivatives. However, it remains unclear whether this effect is mediated by a sequential process, in which geraniol content is first reduced, thereby limiting derivative biosynthesis. Alternatively, the biosynthesis of geraniol derivatives may be directly modulated by these TFs.

**Figure 9:**
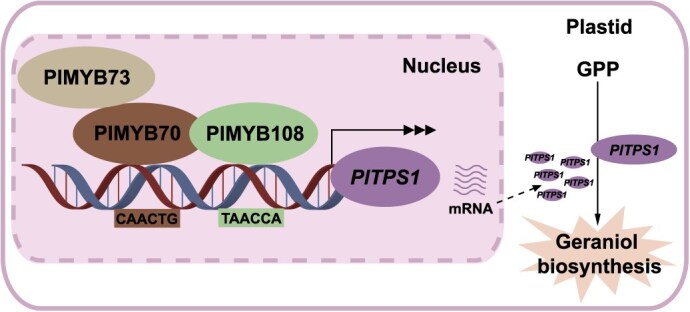
The proposed working model of geraniol biosynthesis in *P. lactiflora* by the PlMYB73–PlMYB70–PlMYB108 transcription complex. PlMYB108 and PlMYB70 directly activate *PlTPS1* expression by binding to the TAACCA and CAACTG motifs, respectively. PlMYB70 interacts with PlMYB73 and PlMYB108, forming a complex. This complex significantly activates *PlTPS1* expression, thereby promoting geraniol biosynthesis.

Geraniol, a key floral scent compound in herbaceous peony, is widely present in fragrant cultivars. Transcriptome analysis and RT-qPCR showed that PlMYB73, PlMYB108, and PlMYB70 were strongly correlated with geraniol emission. These TFs directly participated in geraniol biosynthesis by upregulating *PlTPS1* expression. In the nucleus, PlMYB73, PlMYB108, and PlMYB70 interacted to form the PlMYB73–PlMYB70–PlMYB108 complex, which notably enhanced *PlTPS1* expression and geraniol biosynthesis (Fig. 9). Overall, our findings provide novel insights into the molecular mechanisms governing geraniol biosynthesis in plants, and these insights may facilitate future biotechnological strategies to enhance floral scent emission.

## Materials and methods

### Plant materials and growth conditions

According to our previous research, the cultivar *P. lactiflora* ‘WHLY’ was selected as the study material [43]. Flowers were collected from five developmental stages (S1–S5) as defined previously [15]: S1 (bud stage), when bracts opened; S2 (half-opened stage), with flowers semiopen; S3 (full-opened stage), characterized by fully expanded petals; S4 (postopening), 2 days postbloom with early senescence; and S5 (senescence), 6 days postbloom with petal abscission. Additionally, plants at the S3 stage were separated into seven parts: pistil, stamen, torus, calyx, petal, stem, and leaf. *Nicotiana benthamiana* was used for subcellular localization, Dual-LUC, and LCI assays, while *N. tabacum* was used for stable transformation experiments.

### Analysis of volatile compounds

Sample weights, internal standard content, HS-SPME method, and GC–MS conditions were consistent with those described by Zhao *et al*. [43]. The NIST 14 library was used to determine volatile compounds. The content of volatile terpenes was calculated using the internal standard method, following the formula described in the previous study [43].

### RNA extraction, cDNA synthesis, and RT-qPCR analysis

The methods for RNA extraction, cDNA synthesis, and RT-qPCR analysis were consistent with the previous study [43]. Candidate genes were screened based on the transcriptome data (accession number SRP287892). The primers used are detailed in Table S7. Gene accession numbers are provided in Table S1.

### Gene cloning and sequence analysis

The coding sequences (CDSs) of *PlMYB73*, *PlMYB108*, and *PlMYB70* were amplified using specific primers listed in Table S7. The methods for phylogenetic tree construction and sequence alignment were consistent with the previous study [15]. Table S1 provides the protein accession numbers.

### Subcellular localization

The CDSs of *PlMYB73*, *PlMYB108*, and *PlMYB70* (excluding termination codons) were cloned and ligated into the pCAMBIA2300-GFP vector. The primers used are detailed in Table S7. The recombinant plasmids and empty pCAMBIA2300-GFP vector were used to transform *Agrobacterium tumefaciens* GV3101. One-month-old tobacco leaves were injected with *A. tumefaciens* strains, incubated in darkness for 1 day, and then grown under normal conditions for 2 days. Fluorescence analysis of infected leaves was performed using a laser scanning confocal microscope (DMi8, Leica Microsystems, Wetzlar, Germany). Tobacco leaves were injected with the empty pCAMBIA2300-GFP vector served as controls. DAPI was used as a fluorescent stain to indicate nuclear localization, and the staining process was performed as described by Yang *et al*. [79].

### Transient silencing and overexpression experiments

For the VIGS assay, non-coding region fragments (300 bp) of *PlMYB73*, *PlMYB108*, and *PlMYB70* were cloned and ligated into the pTRV2 vector to generate pTRV2-*PlMYB73/108/70*. The recombinant plasmids, empty pTRV2, and pTRV1 were introduced into *A. tumefaciens* strain GV3101. Positive colonies were selected on LB medium containing 50 μg·ml^−1^ kanamycin and 50 μg·ml^−1^ rifampicin. Verified colonies were cultured at 28°C with shaking until the OD_600_ reached 0.6–0.8. Bacterial cells were pelleted by centrifugation and resuspended in infiltration buffer (10 mM MES, 0.1 mM acetosyringone, 10 mM MgCl₂, pH 5.6) to a final OD_600_ of 0.6. Equal volumes (1:1) of pTRV1 and pTRV2 (or pTRV2-*PlMYB73/108/70*) suspensions were mixed and incubated in darkness at 28°C for 3 h. Petal discs (1.5 cm diameter) from the central region of S1-stage flowers were vacuum-infiltrated (0.9 MPa, 15 min) with the bacterial suspension, rinsed with deionized water, and incubated in darkness for 1 day followed by 2 days under normal growth conditions [80].

For transient overexpression, the CDSs of *PlMYB73*, *PlMYB108*, and *PlMYB70* were cloned and ligated into the pCAMBIA1300 vector to generate p1300-*PlMYB73/108/70*. *Agrobacterium*-mediated transformation, bacterial resuspension, and petal disc treatment followed the same protocol as the VIGS assay.

For co-silencing or co-overexpression experiments, bacterial suspensions of pTRV2-*PlMYB73/108/70* or p1300-*PlMYB73/108/70* were mixed at equal volumes (1:1 for dual-gene combinations or 1:1:1 for triple-gene combinations) to generate the respective co-transformation groups.

Positive samples of VIGS or overexpression experiments were confirmed by PCR and RT-qPCR. *GAPDH* served as the internal reference gene. Primers for this section are listed in Table S7. Positive samples were subjected to HS-SPME-GC–MS for volatile terpenoid analysis. Qualitative and quantitative analyses were performed as previously described [43].

### DNA extraction, promoter cloning, and *cis*-acting element analysis

DNA was extracted using a Plant Genomic DNA Kit (DP305, Tiangen, Beijing, China). The promoter of *PlTPS1* was amplified using a series of specific primers (SP primers) with high annealing temperature using the Genome Walking Kit (6108, Takara, Dalian, China) [81]. The *PlTPS1pro* sequence was analyzed via the PlantCARE database. Primers are listed in Table S7, and the *PlTPS1pro* sequence is shown in Fig. S8.

### Y1H assay

A 1776-bp *PlTPS1pro* sequence was cloned from genomic DNA and inserted into the pHIS2 vector as bait. The truncated versions of the *PlTPS1pro*, namely P1 (1365 bp), P2 (800 bp), and P3 (312 bp), were also inserted into the pHIS2 vector. The CDSs of *PlMYB73*, *PlMYB108*, and *PlMYB70* were cloned and ligated into the pGADT7 vector. Primers for these constructs are listed in Table S7, and methods were performed as described by Yang *et al*. [79]. The constructs were co-transformed into yeast strain Y187 and screened on a selective medium (SD/−Leu/−Trp/-His) containing 3-AT. A 3-AT gradient assay (0, 20, 40, 60, 80, 100 mM) was conducted to determine the minimal concentration for suppressing background yeast growth. Yeast cells containing p53-pHIS2 + pGADT7-p53 served as the positive control.

### Dual-LUC reporter assay

The CDSs of *PlMYB73*, *PlMYB108*, and *PlMYB70* were cloned and ligated into the pGreenII 62-SK effector vector, and the promoter region of *PlTPS1* was cloned and ligated into the pGreenII 0800-LUC reporter vector. Table S7 lists the primers used. Tobacco leaves were infiltrated as described by Yang *et al*. [79]. Two days post-injection, chemiluminescent signals of LUC and REN were quantified using a Dual-LUC Reporter Gene Assay Kit (Yesen, Shanghai, China). Additionally, fluorescence was visualized using a multispectral dynamic fluorescence microscopy imaging system (PlantView, Guangzhou, China) after the leaves were sprayed with 1 mM D-luciferin potassium salt.

### EMSA

The CDSs of *PlMYB108* and *PlMYB70* were cloned and ligated into the pET32a vector to generate the His-tagged PlMYB108 and PlMYB70 proteins. Table S7 lists the primers used. Induction and purification of His-PlMYB108 and His-PlMYB70 proteins were performed as described by Zhao *et al*. [15]. Three 36-bp probes, each containing one of the TAACCA, CAACTG, or CAACCA motifs within the *PlTPS1pro*, were synthesized and biotin-labeled by General Biol. (Anhui, China). The competition probes were unlabeled DNA fragments, and the mutation probes contained mutated TAACCA and CAACTG motifs. The assays were performed according to the manufacturer’s instructions for the Chemiluminescent EMSA Kit (Beyotime, Shanghai, China). The imaging was performed via a chemiluminescence imaging system (ChemiDoc, Bio-Rad, CA, USA).

### Heterologous stable transformation in tobacco

The CDSs of *PlMYB108* and *PlMYB70* were cloned and ligated into the pCAMBIA1300 vector that harbored a hygromycin-resistant gene. The stable transformation method was performed as described by Zhao *et al*. [15]. OE lines were screened via DNA detection and RT-qPCR analysis, with *EF1α* serving as the internal reference gene. Table S7 shows the primers used. Both WT and OE flowers were sampled for HS-SPME-GC–MS analysis, and the content of terpenes was calculated by the internal standard method [43].

### Y2H assay

The CDSs of *PlMYB108* and *PlMYB70* were cloned and ligated into the pGBKT7 vector. The CDSs of *PlMYB73* and *PlMYB108* were cloned and ligated into the pGADT7 vector. Table S7 shows the primers used. Then the corresponding combinations were co-transformed into yeast strain Y2HGold, and screened on a selective medium (SD/−Trp-Leu-His-Ade) containing 100 ng·ml^−1^ AbA and 40 μg·ml^−1^ X-α-Gal. Yeast cells harboring pGADT7-T + pGBKT7-p53 served as the positive control.

### BiFC assay

The CDSs without termination codon of *PlMYB108* and *PlMYB70* were cloned and ligated into the pSPYCE-35S vector. The CDSs without termination codon of *PlMYB73* and *PlMYB108* were cloned and ligated into the pSPYNE-35S vector. Table S7 shows the primers used. Tobacco leaves were infiltrated as described by Yang *et al*. [79]. Two days after inoculation, YFP signals were detected using a laser scanning confocal microscope (DMi8, Leica Microsystems, Wetzlar, Germany). DAPI was used as a fluorescent stain to indicate nuclear localization, and the staining process was performed as described by Yang *et al*. [79].

### LCI assay

The CDSs without termination codon of *PlMYB73* and *PlMYB108* were cloned and ligated into the nLUC vector, while the CDSs without termination codon of *PlMYB70* and *PlMYB73* were cloned and ligated into the cLUC vector. Table S7 lists the primers used. The method was performed as described by Yang *et al*. [79]. Luciferase luminescence was detected as described in the previous Dual-LUC assay.

### Data analysis

Each sample was analyzed in three independent replicates. Data are presented as means ± SD. Heatmaps were generated using Metware Cloud (https://cloud.metware.cn/#/home). Statistical significance was determined by Duncan’s multiple range test (multiple groups) and independent samples *t*-test (two groups). In this research, the independent samples *t*-test was only used for comparisons between TRV2 and TRV2-*PlMYB73/108/70*, or p1300 and p1300-*PlMYB73/108/70*.

## Supplementary Material

Web_Material_uhaf141

## Data Availability

The data supporting the findings of this study are available within the paper figures and the Supplementary Information.
