# Supplementary material for: The PlMYB73–PlMYB70–PlMYB108 complex regulates PlTPS1 to promote geraniol biosynthesis in Paeonia lactiflora
Source: Hortic Res. 2025 May 29;12(8):uhaf141. doi: 10.1093/hr/uhaf141 (PMC12282130; doi:10.1093/hr/uhaf141)
Supplement: Web_Material_uhaf141 [file web_material_uhaf141.zip › Supplementary figures.docx]

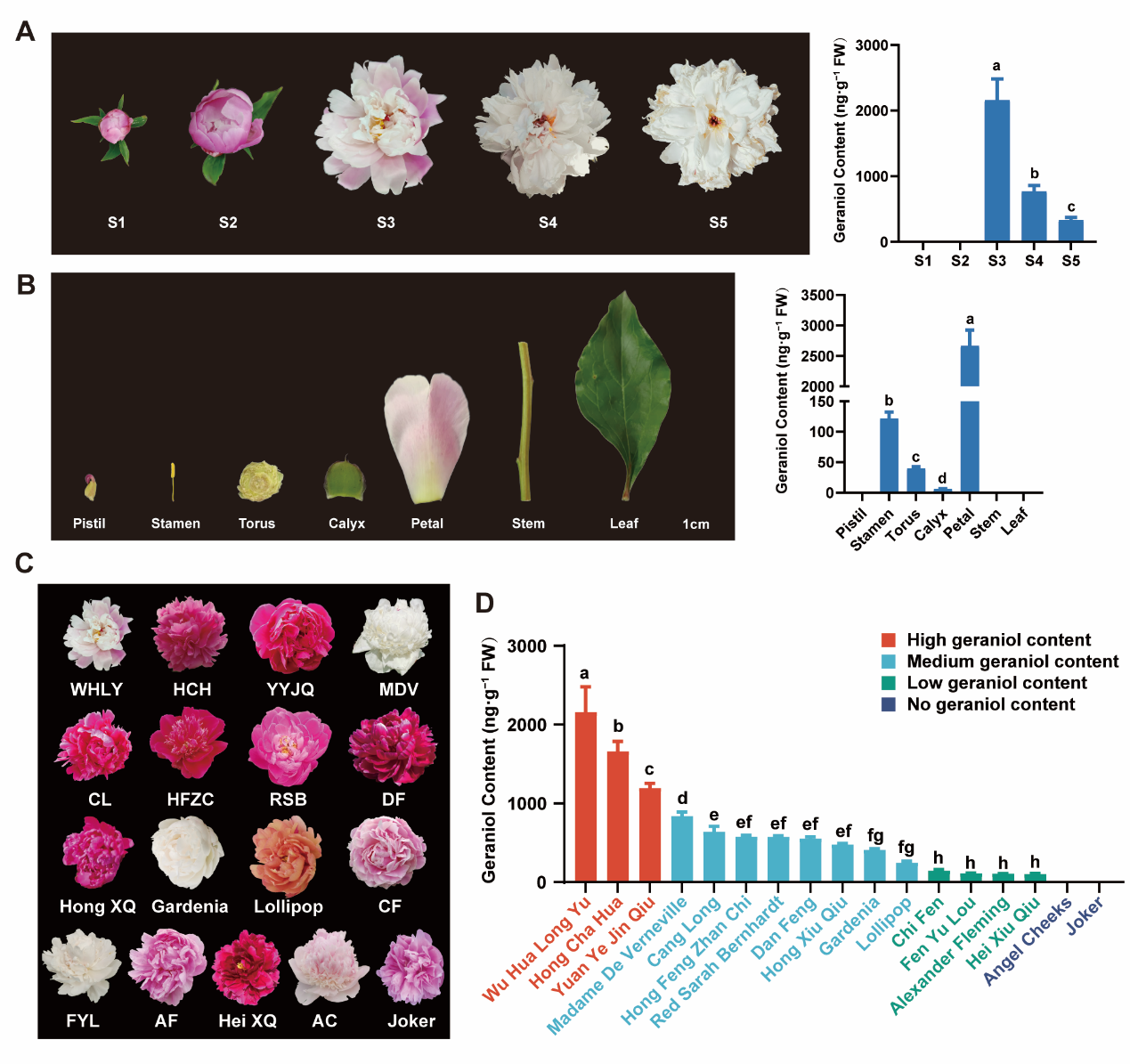


**Figure S1.** Geraniol release patterns in different stages, organs, and cultivars. (A) Developmental stages of ‘WHLY’ and geraniol content in different stages. (B) Organs from the S3 stage of ‘WHLY’ and geraniol content in different organs. (C) Phenotypes of 17 herbaceous peony cultivars. (D) Geraniol content of 17 cultivars. Geraniol content of ‘WHLY’ and cDNA used for RT-qPCR were prepared in 2024. Geraniol content of the remaining cultivars as well as cDNA used for RT-qPCR was referenced from Zhao et al., 2023 [42]. Data are presented as mean ± SD from three biological replicates. Different lowercase letters above columns indicate statistically significant differences (*P* < 0.05).


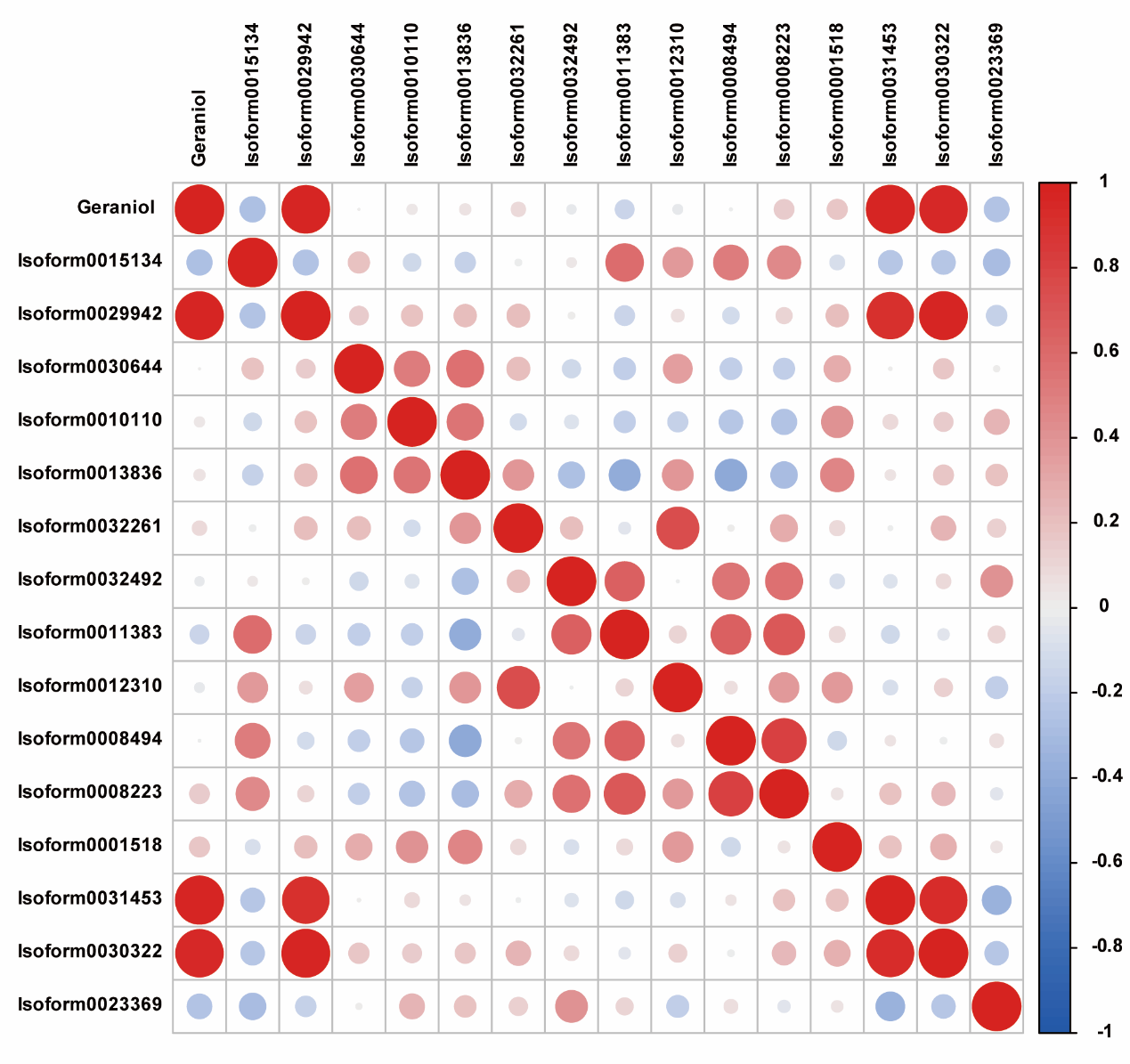


**Figure S2.** Heatmap of correlation between geraniol content and 15 candidate MYB TFs. Color gradients range from blue to red (coefficients from -1 to 1). Larger circles indicate stronger correlations.


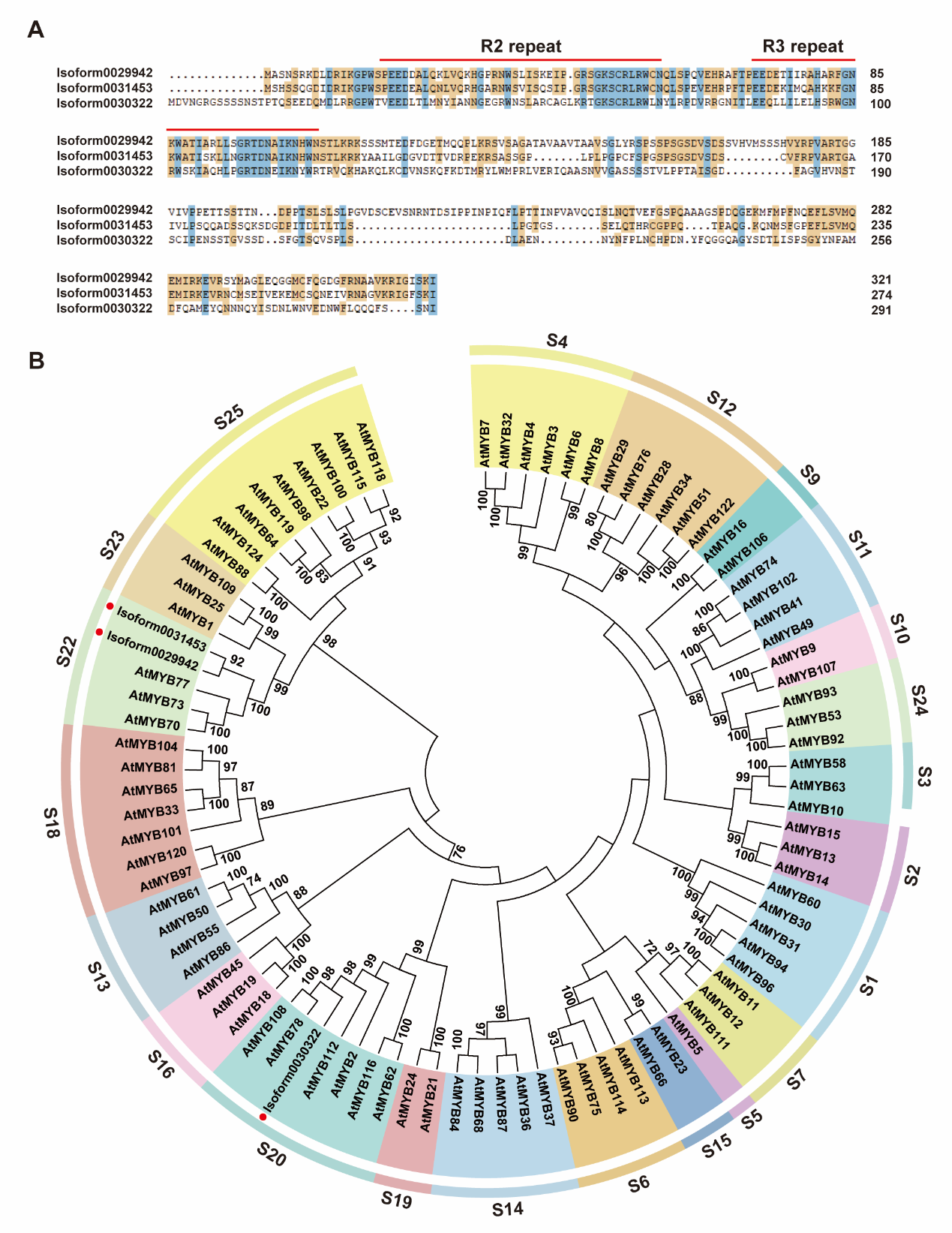
 **Figure S3.** Bioinformatics analysis of three *P. lactiflora* R2R3-MYB TFs. (A) Protein alignment among three *P. lactiflora* R2R3-MYB TFs. The conserved R2 and R3 repeat domains are indicated by red lines. (B) Phylogenetic analysis of three *P. lactiflora* R2R3-MYB TFs with *A. thaliana* R2R3-MYB TFs. The neighbor-joining tree was generated by MEGA7 using the p-distance model with 1000 bootstrap replicates. Accession numbers are provided in Table S1.


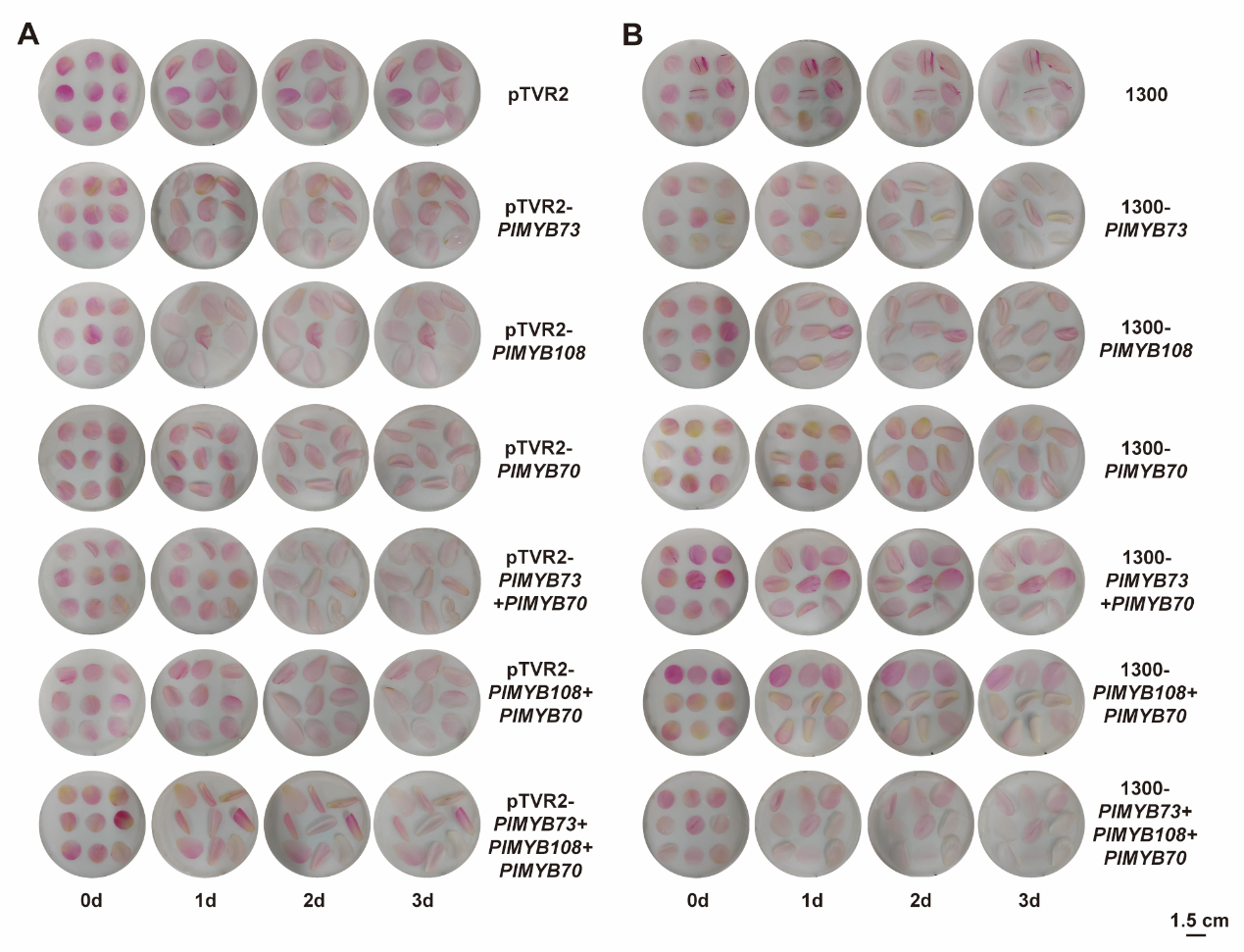


**Figure S4.** Growth status of petal discs during transient silencing and overexpression experiments in *P. lactiflora* ‘WHLY’. (A) Transient silencing experiment. (B) Transient overexpression experiment.


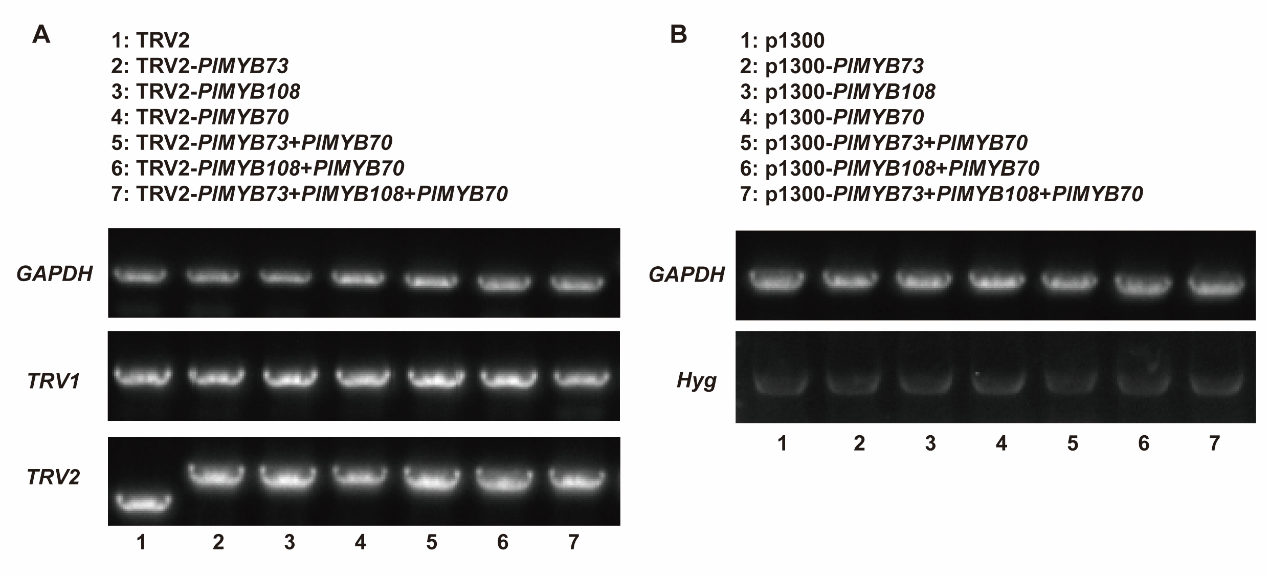
 **Figure S5.** PCR identification of positive petal discs in transient silencing and overexpression experiments. (A) Transient silencing experiment. (B) Transient overexpression experiment. *Hyg*, hygromycin resistance marker.


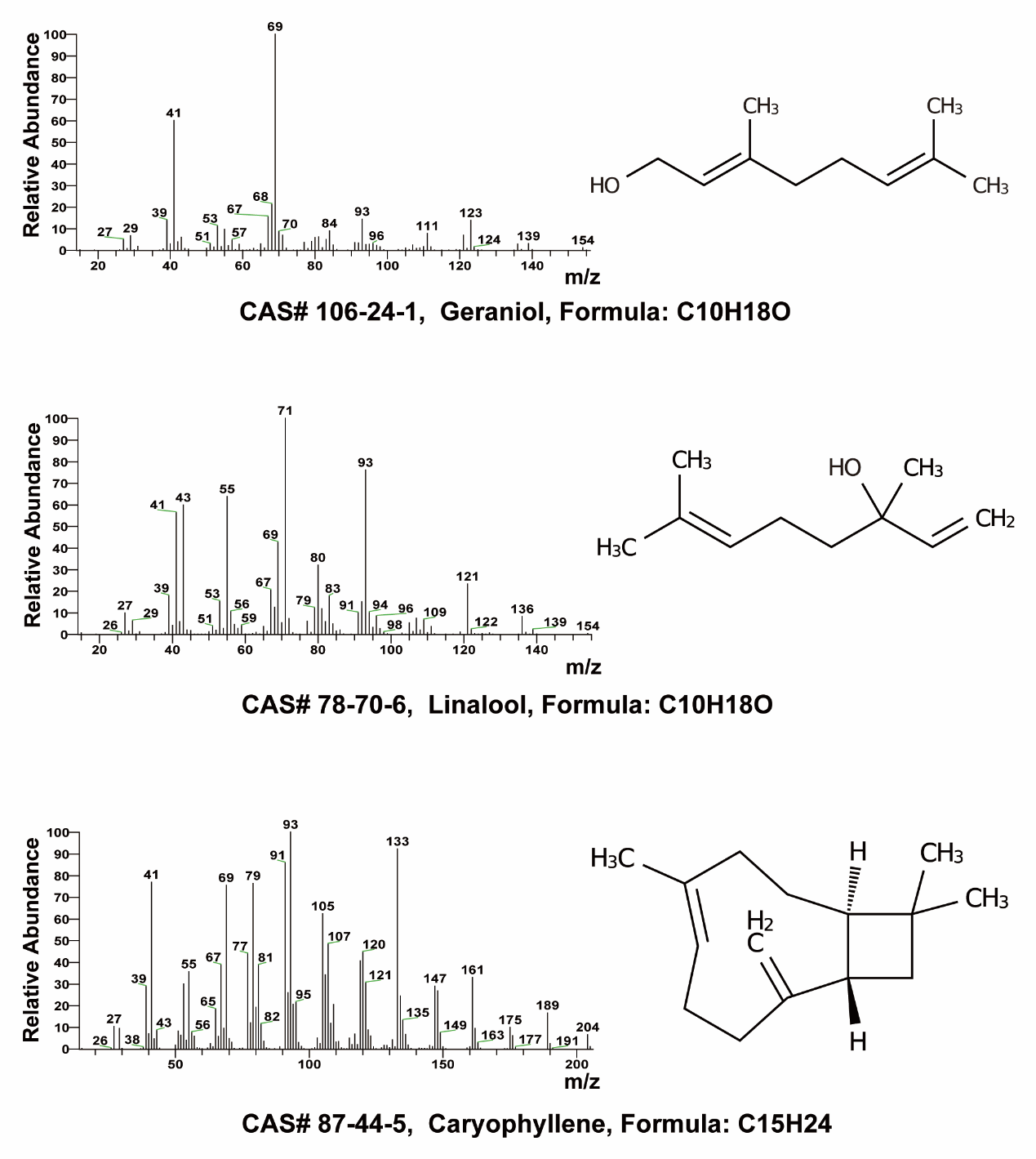


**Figure S6.** Mass spectra and chemical structures of compounds.


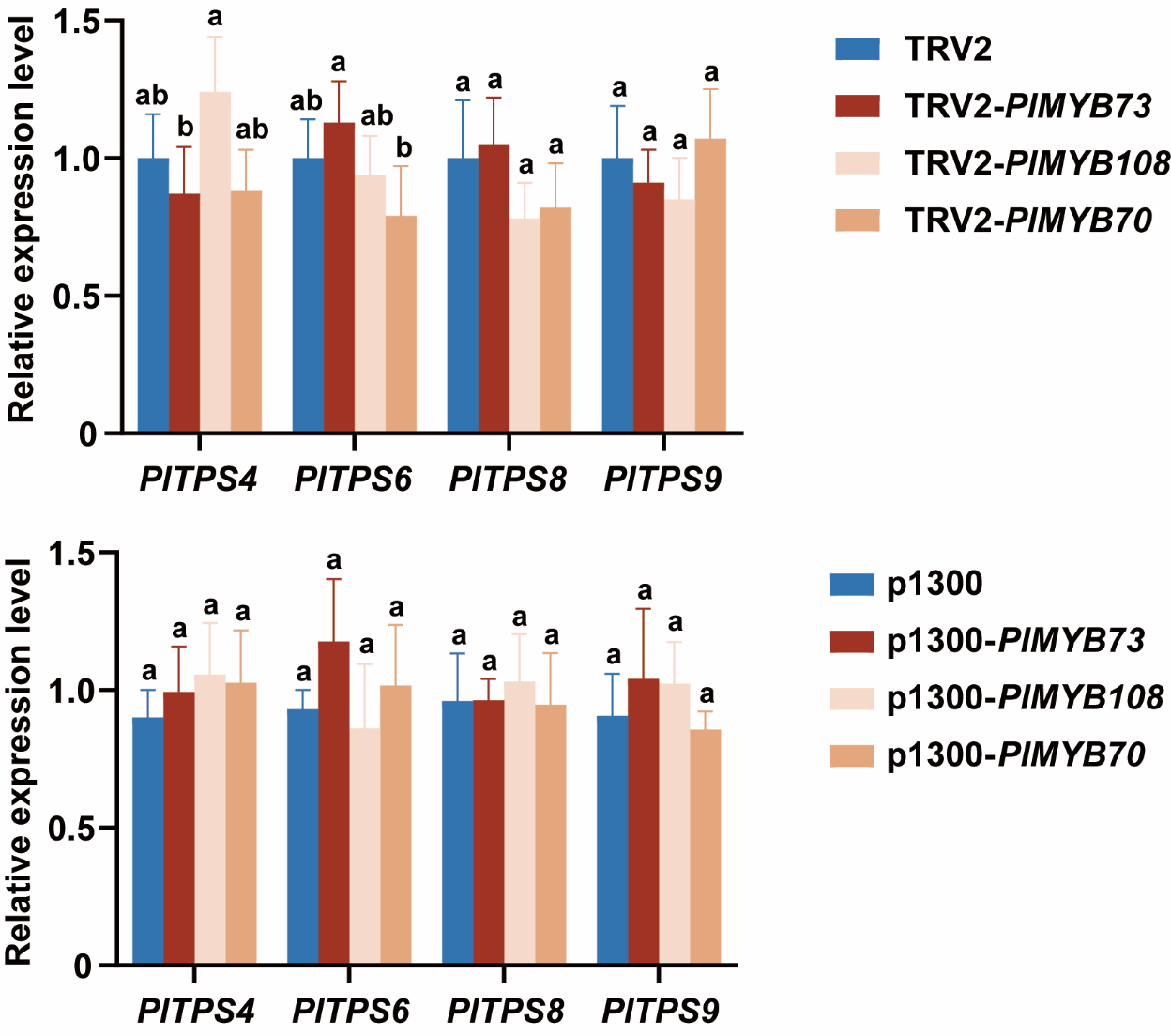


**Figure S7.** RT-qPCR analysis of *PlTPS4/6/8/9* expression levels in transient silencing and overexpression experiments. Data are presented as mean ± SD from three biological replicates. Different lowercase letters above columns indicate statistically significant differences (*P* < 0.05).


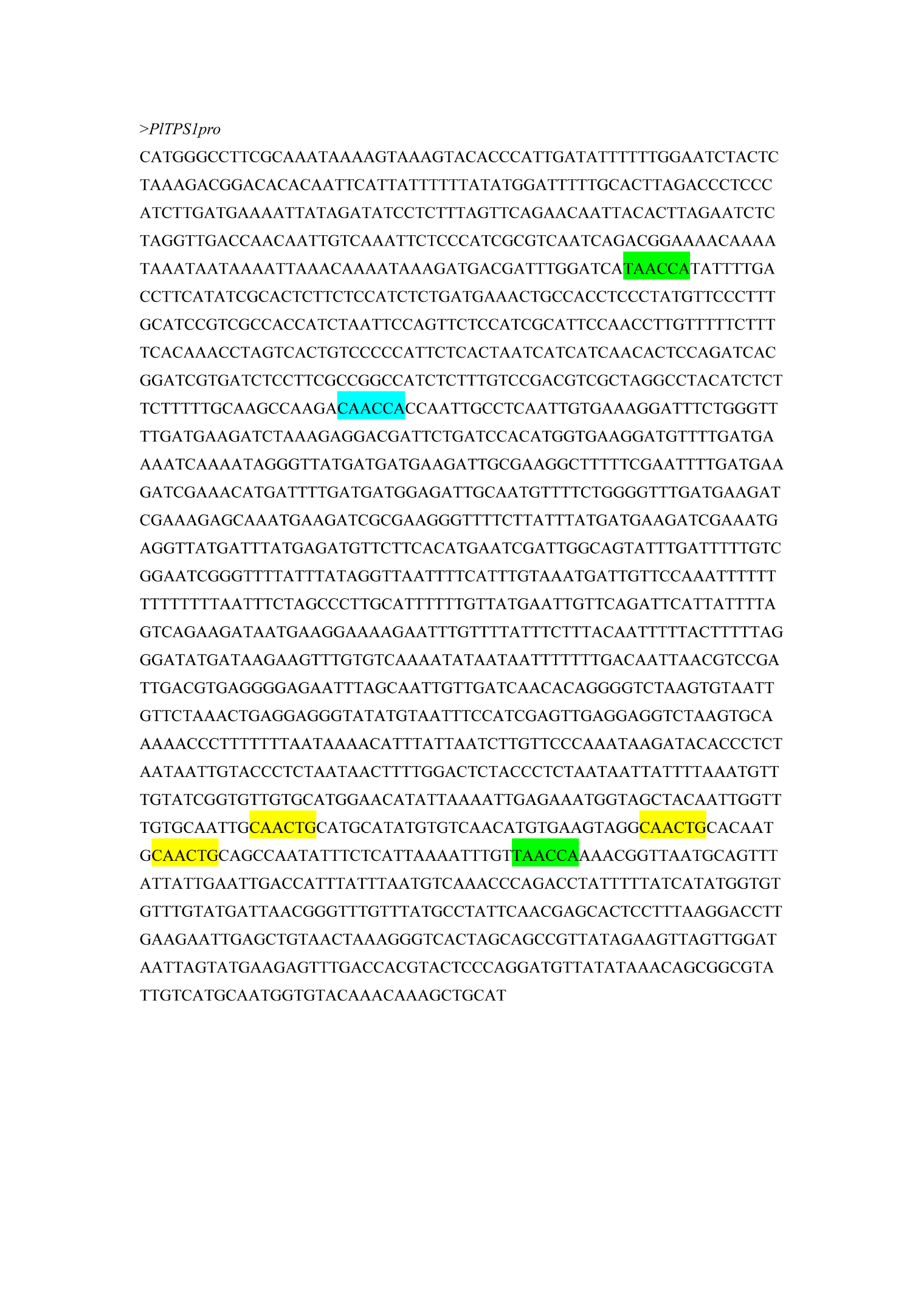


**Figure S8.** Sequence of the *PlTPS1pro* and the identified MYB binding sites.


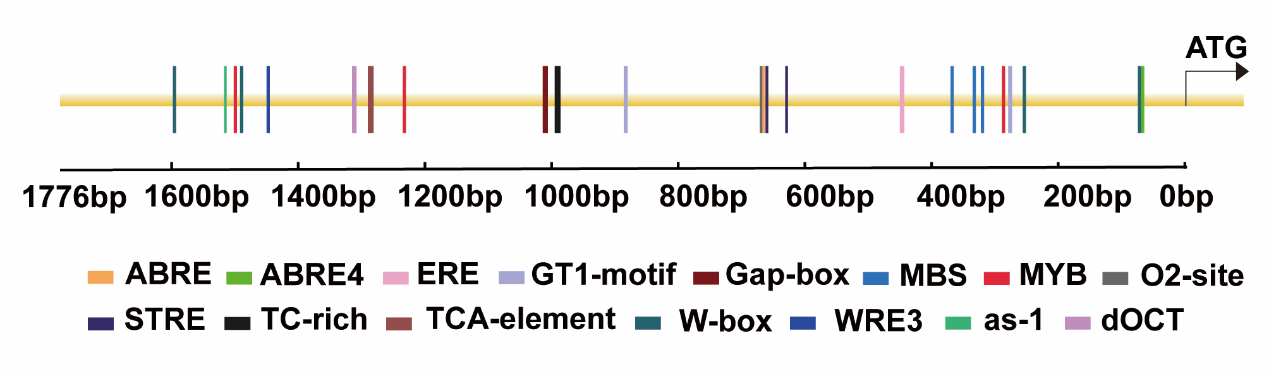


**Figure S9.** Analysis of *cis*-acting elements on the *PlTPS1pro*.


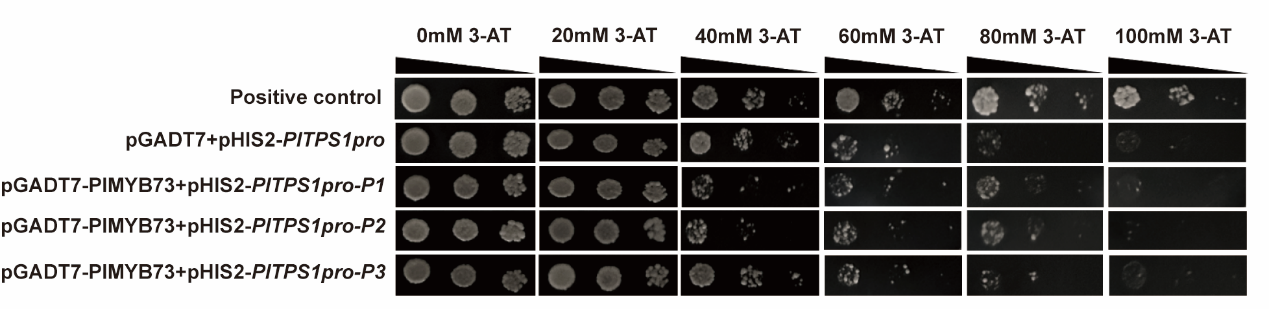


**Figure S10.** Determination of optimal 3-AT concentration for suppressing background growth in the Y1H assay.


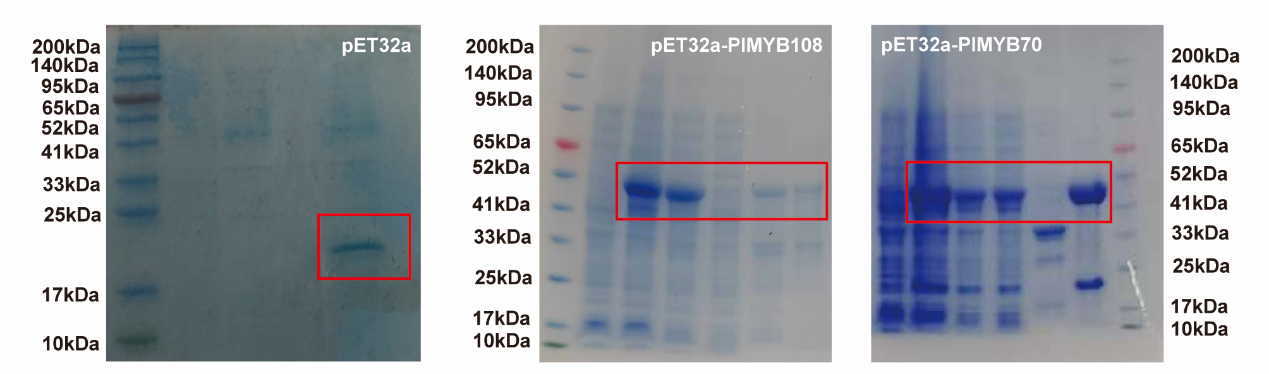


**Figure S11.** SDS-PAGE analysis of empty pET-32a vector, PlMYB108, and PlMYB70 recombinant proteins expressed in Rosetta (DE3). The positions corresponding to the soluble proteins are marked with red boxes.


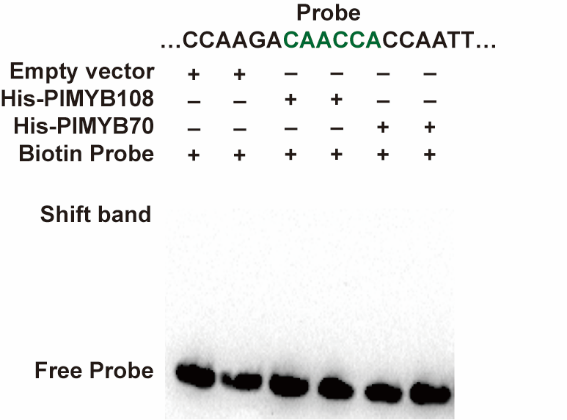


**Figure S12.** Failure of His-PlMYB108 and His-PlMYB70 recombinant proteins to bind the *PlTPS1pro* region containing the CAACCA motif.

**
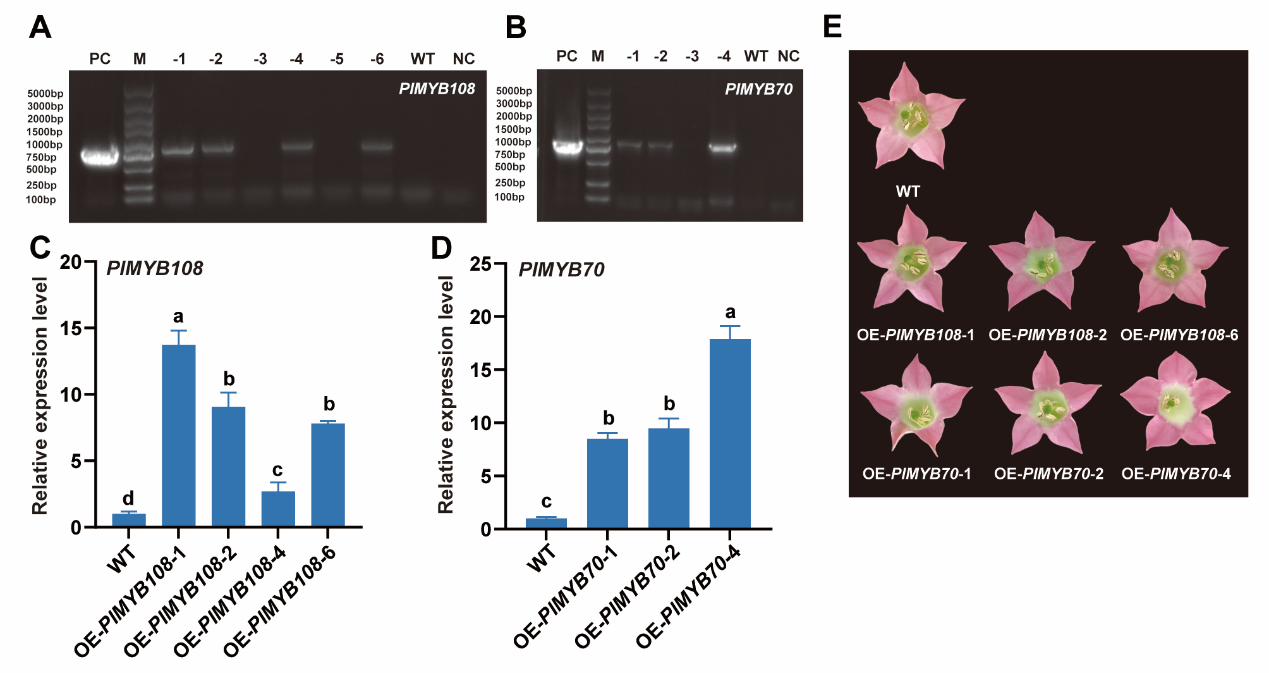
** **Figure S13.** Stable transformation of *PlMYB108* and *PlMYB70* in *N. tabacum*. (A) DNA detection of *PlMYB108* in the WT and OE lines. (B) DNA detection of *PlMYB70* in the WT and OE lines. (C) Relative expression levels of *PlMYB108* in the flowers of WT and OE lines. (D) Relative expression levels of *PlMYB70* in the flowers of WT and OE lines. (E) The phenotypes of the WT and OE lines. *EF1α* was used as the internal reference gene. Data are presented as mean ± SD from three biological replicates. Different lowercase letters above columns indicate statistically significant differences (*P* < 0.05).
